# Supplementary figures and images for: Autoregulatory control of microtubule binding in doublecortin-like kinase 1
Source: eLife. 2021 Jul 26;10:e60126. doi: 10.7554/eLife.60126 (PMC8352597; doi:10.7554/eLife.60126)

Figure 1-source data

B

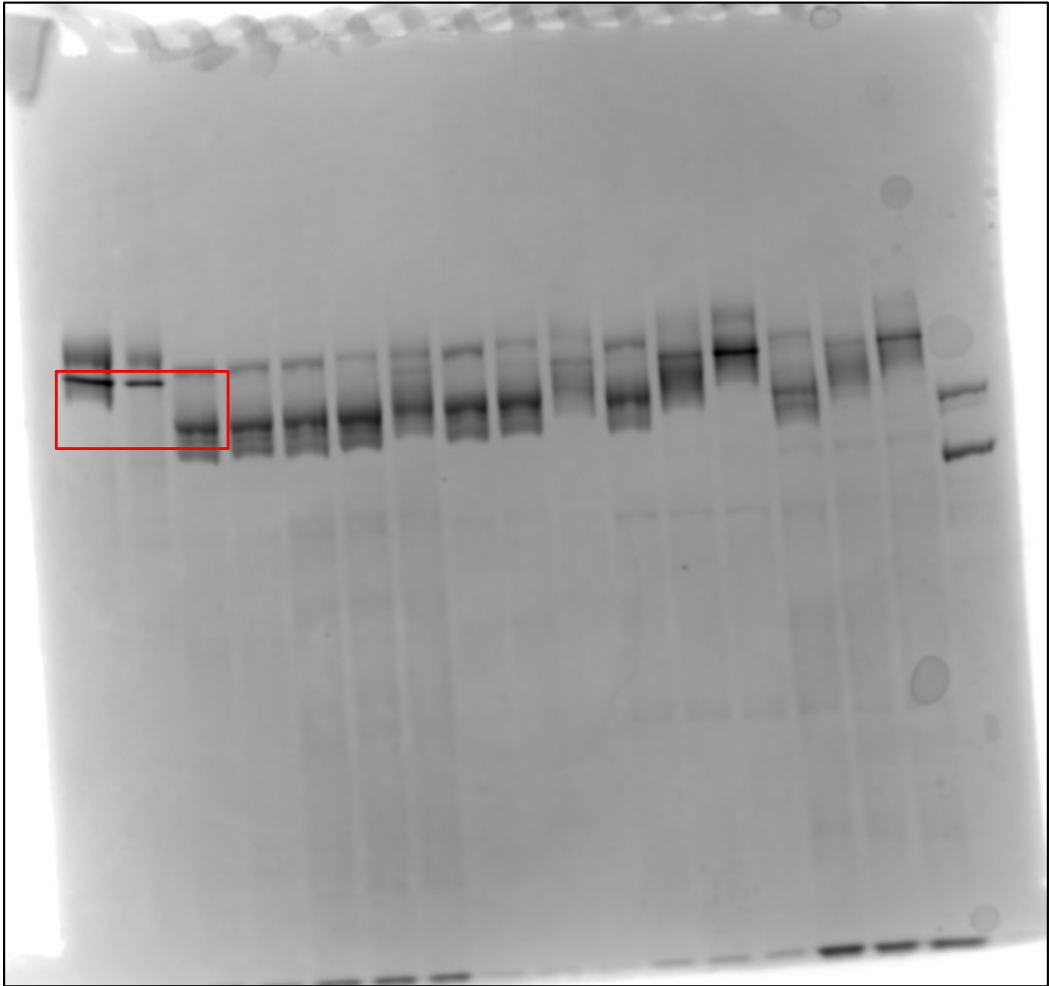

E

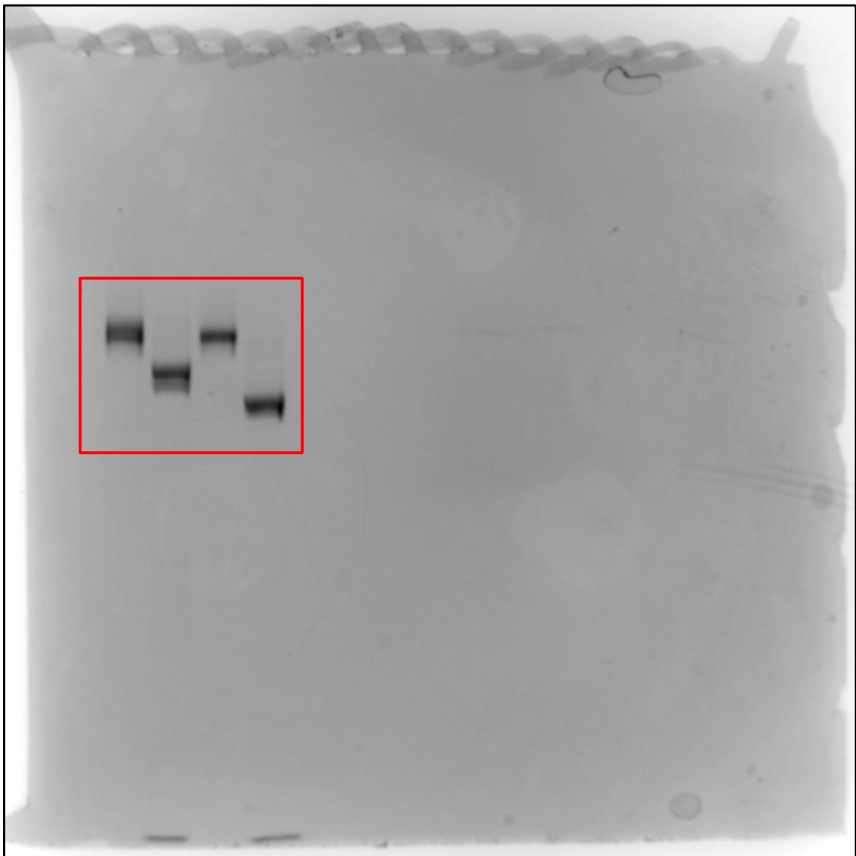

Supplement: Figure 1—source data 1. — The red box indicates how the gel was cropped. [file elife-60126-fig1-data1.pdf]

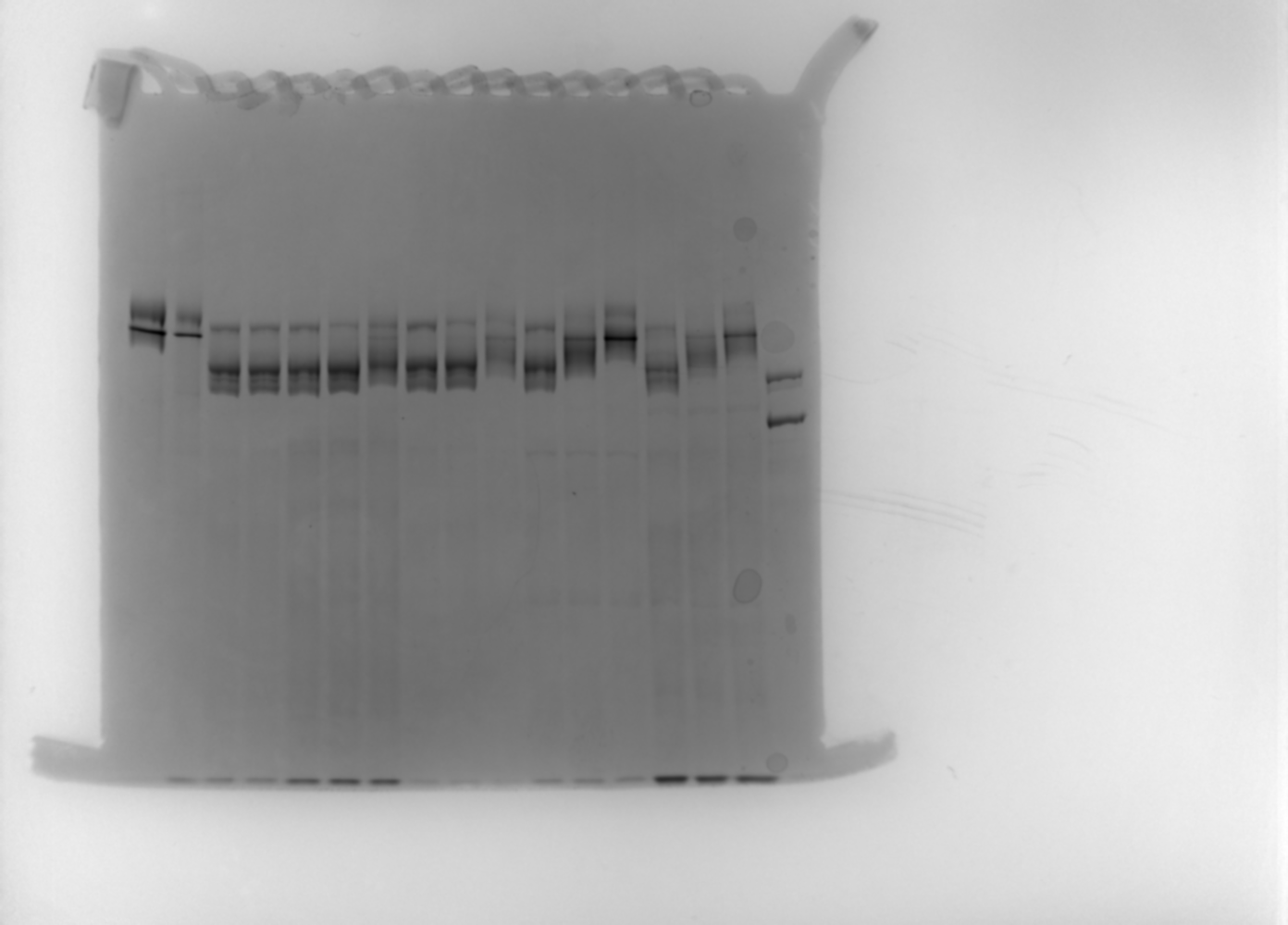

Supplement: Figure 1—source data 2. [file elife-60126-fig1-data2.zip › Figure 1-source data uncropped gels/Figure 1-source data 2.tif]

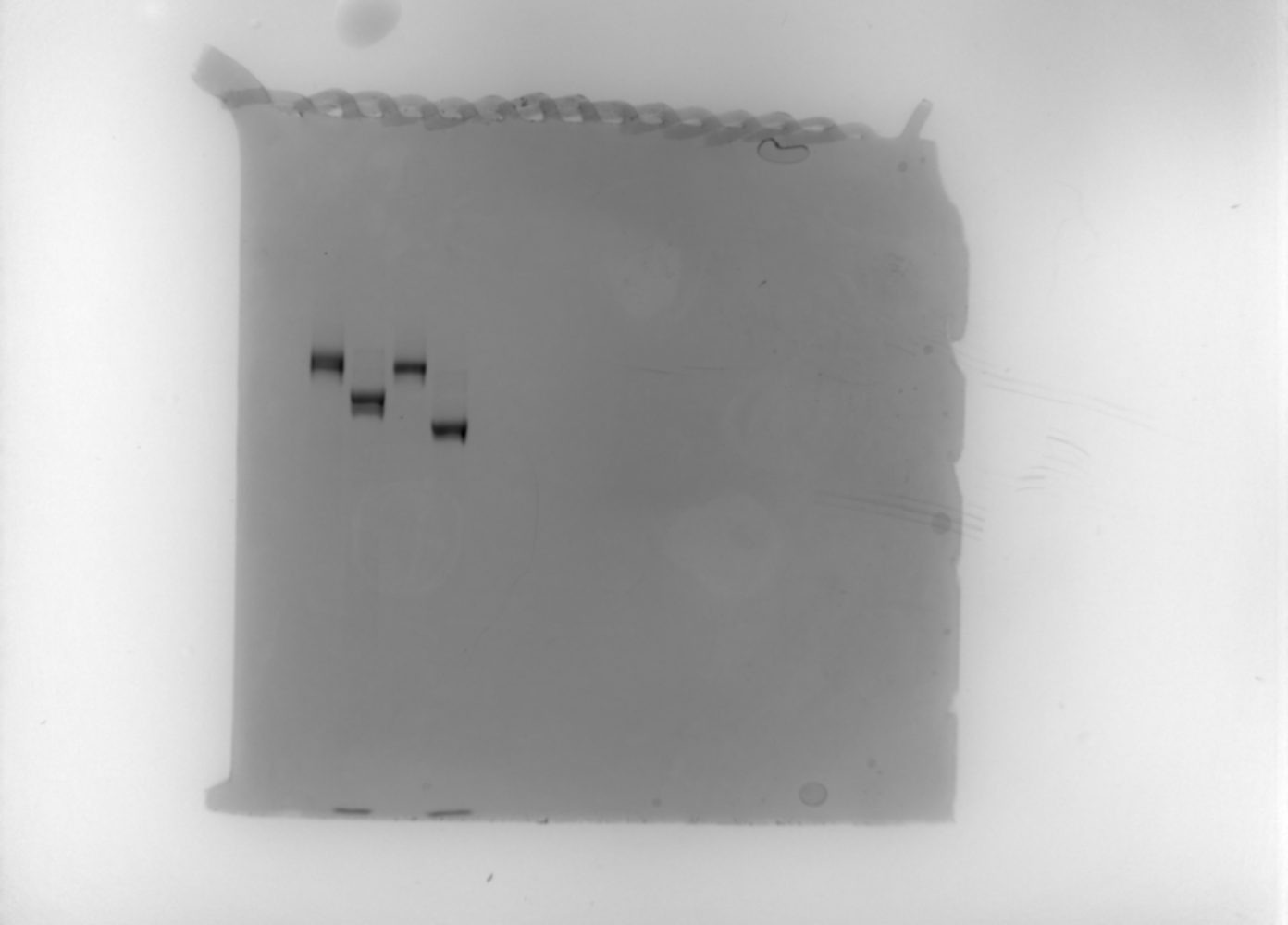

Supplement: Figure 1—source data 2. [file elife-60126-fig1-data2.zip › Figure 1-source data uncropped gels/Figure 1-source data 3.tif]

Figure 1-figure supplement 1-source data

WT

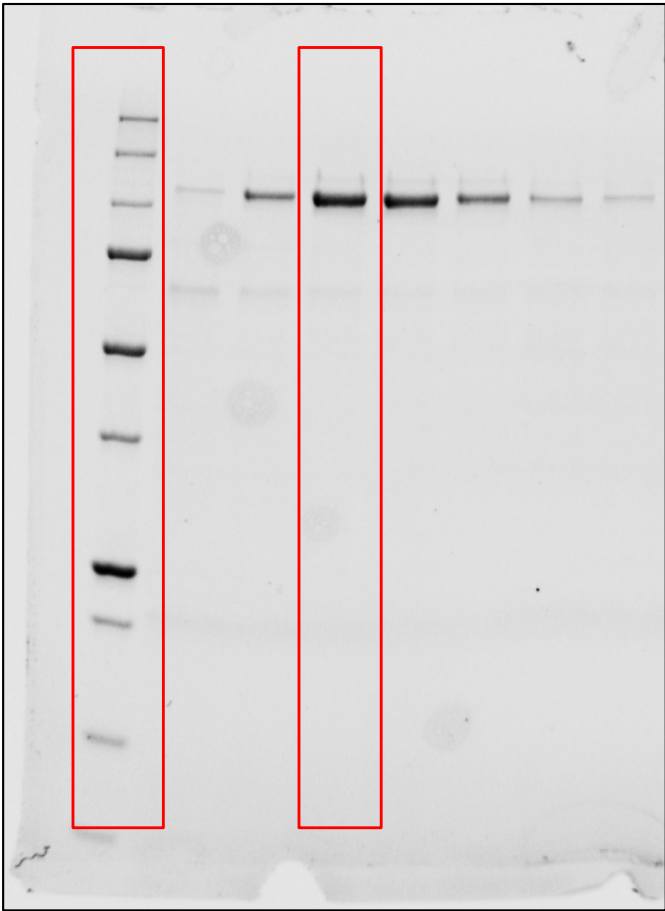

D511N

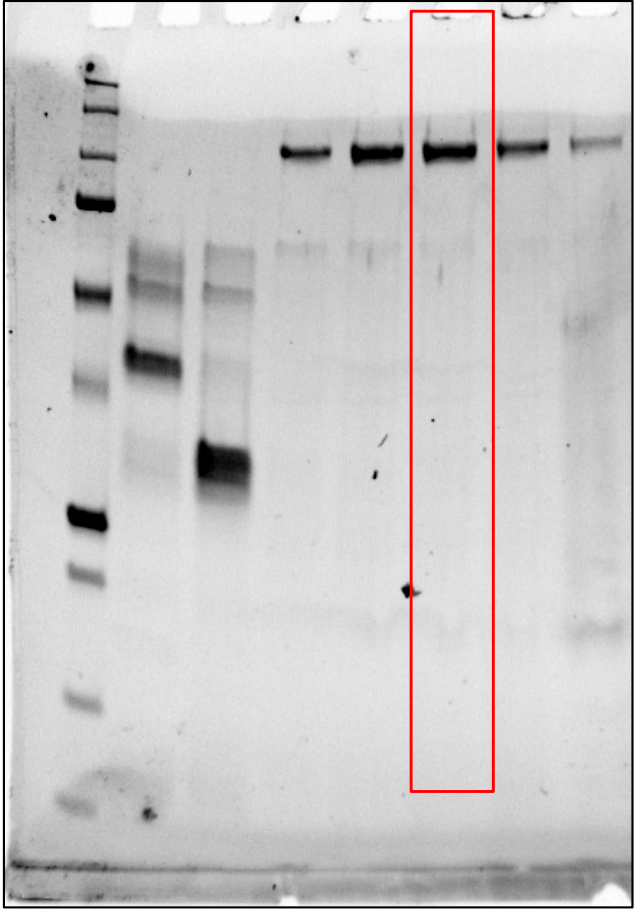

T687A

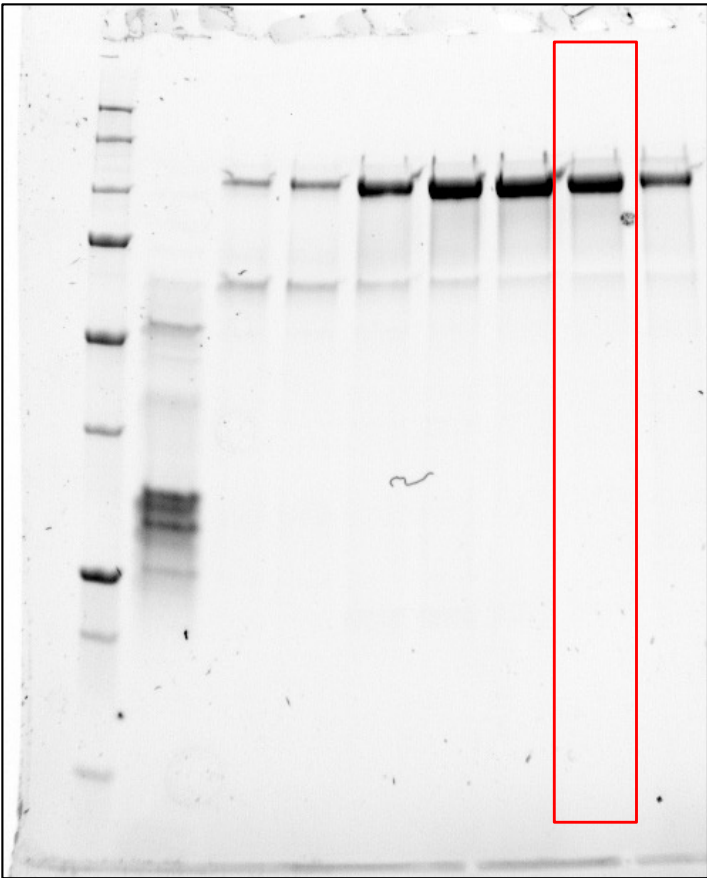

T688A

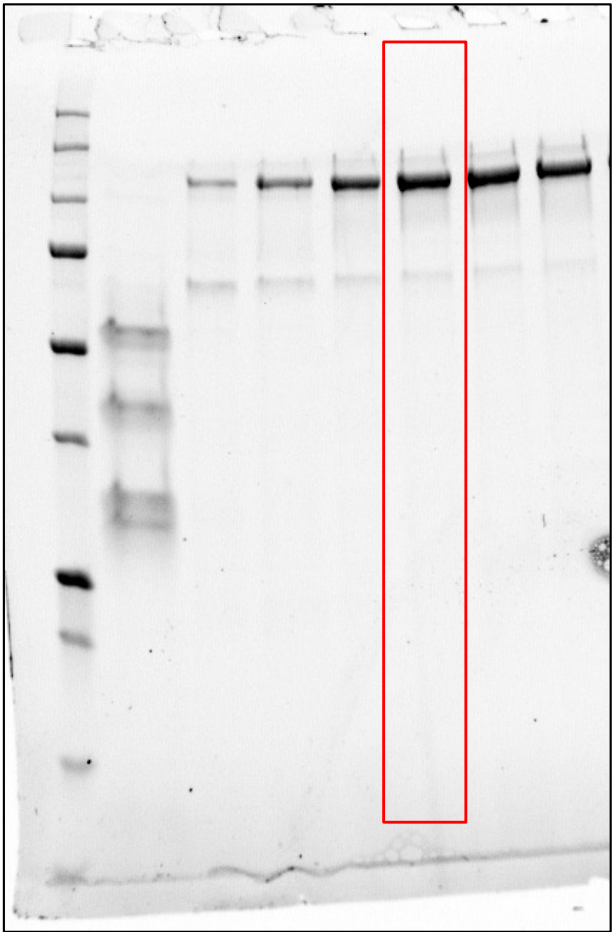

Figure 1-figure supplement 1-source data

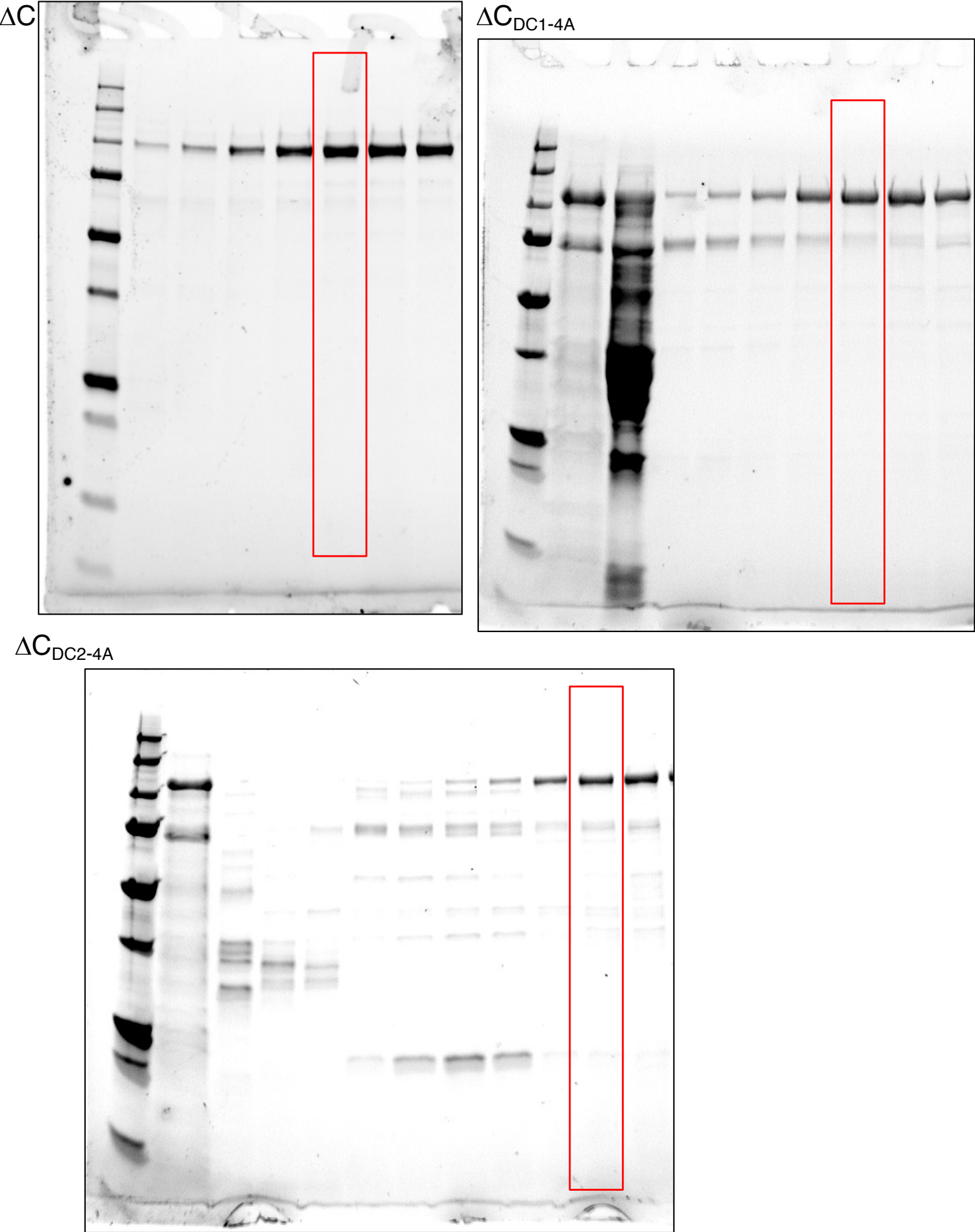

Supplement: Figure 1—figure supplement 1—source data 1. — The red box indicates how the gel was cropped. [file elife-60126-fig1-figsupp1-data1.pdf]

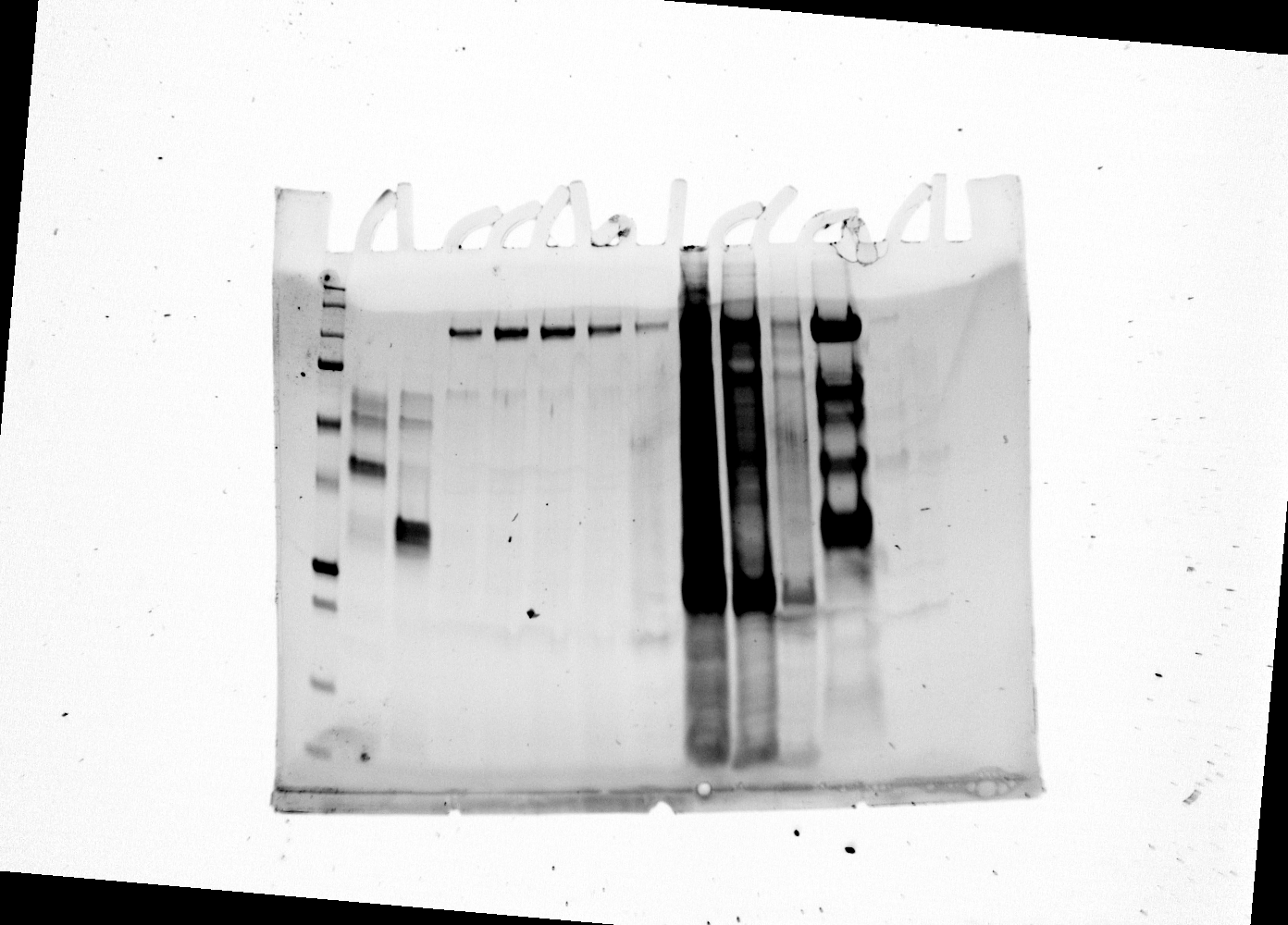

Supplement: Figure 1—figure supplement 1—source data 2. [file elife-60126-fig1-figsupp1-data2.zip › Figure 1-figure supplement 1-source data uncropped gels/Figure 1-figure supplement 1-source data 2.tif]

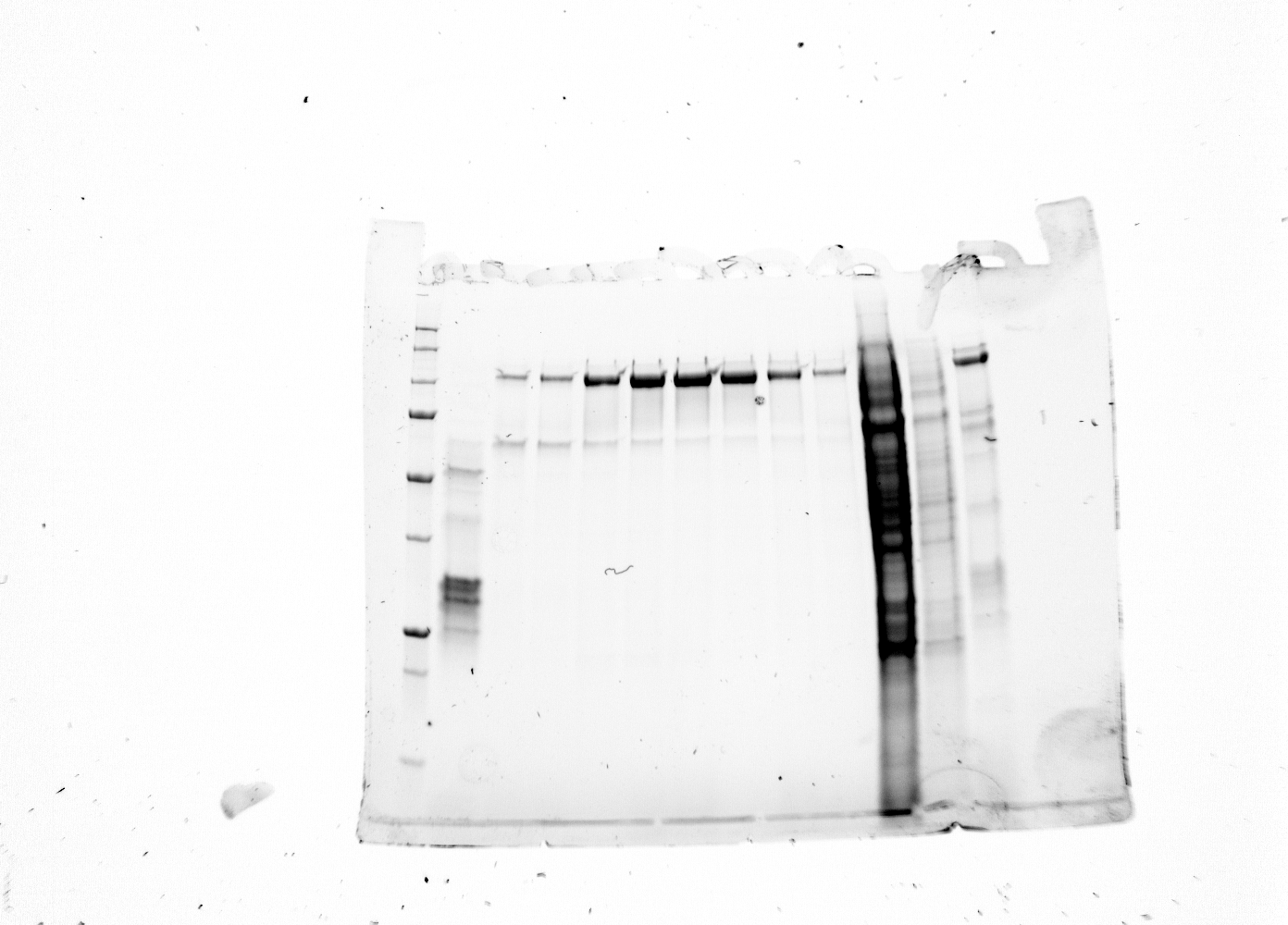

Supplement: Figure 1—figure supplement 1—source data 2. [file elife-60126-fig1-figsupp1-data2.zip › Figure 1-figure supplement 1-source data uncropped gels/Figure 1-figure supplement 1-source data 3.tif]

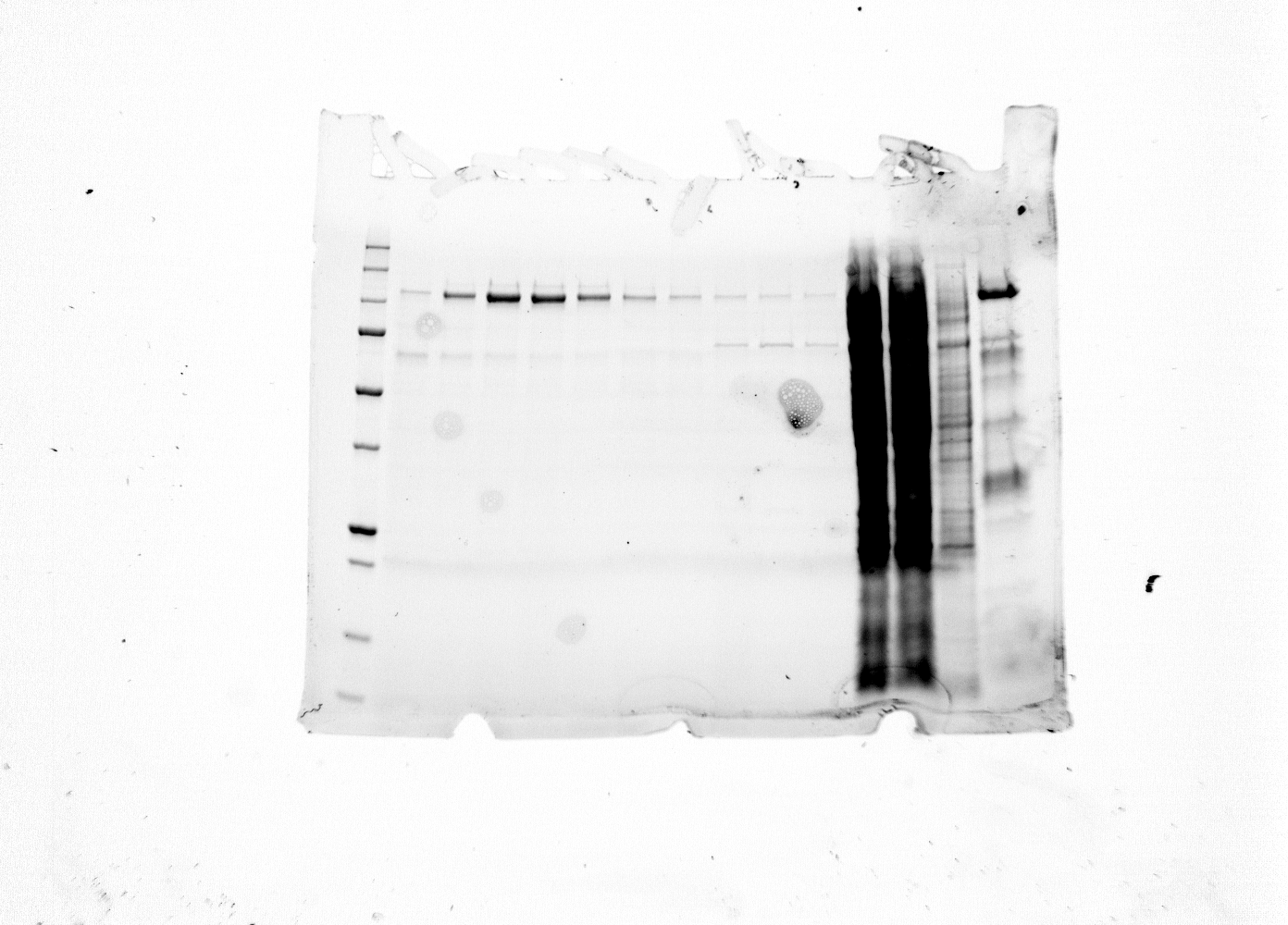

Supplement: Figure 1—figure supplement 1—source data 2. [file elife-60126-fig1-figsupp1-data2.zip › Figure 1-figure supplement 1-source data uncropped gels/Figure 1-figure supplement 1-source data 1.tif]

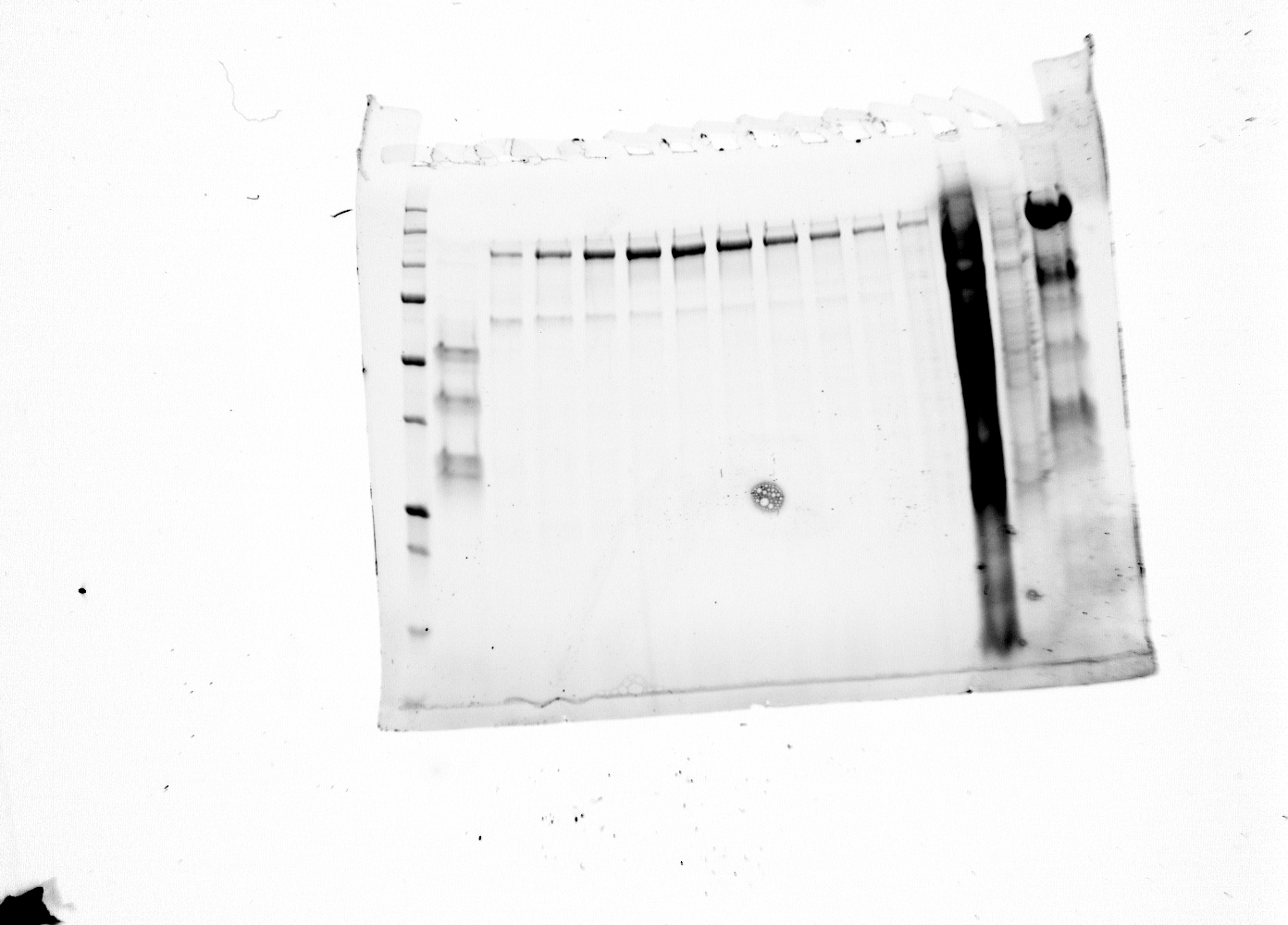

Supplement: Figure 1—figure supplement 1—source data 2. [file elife-60126-fig1-figsupp1-data2.zip › Figure 1-figure supplement 1-source data uncropped gels/Figure 1-figure supplement 1-source data 4.tif]

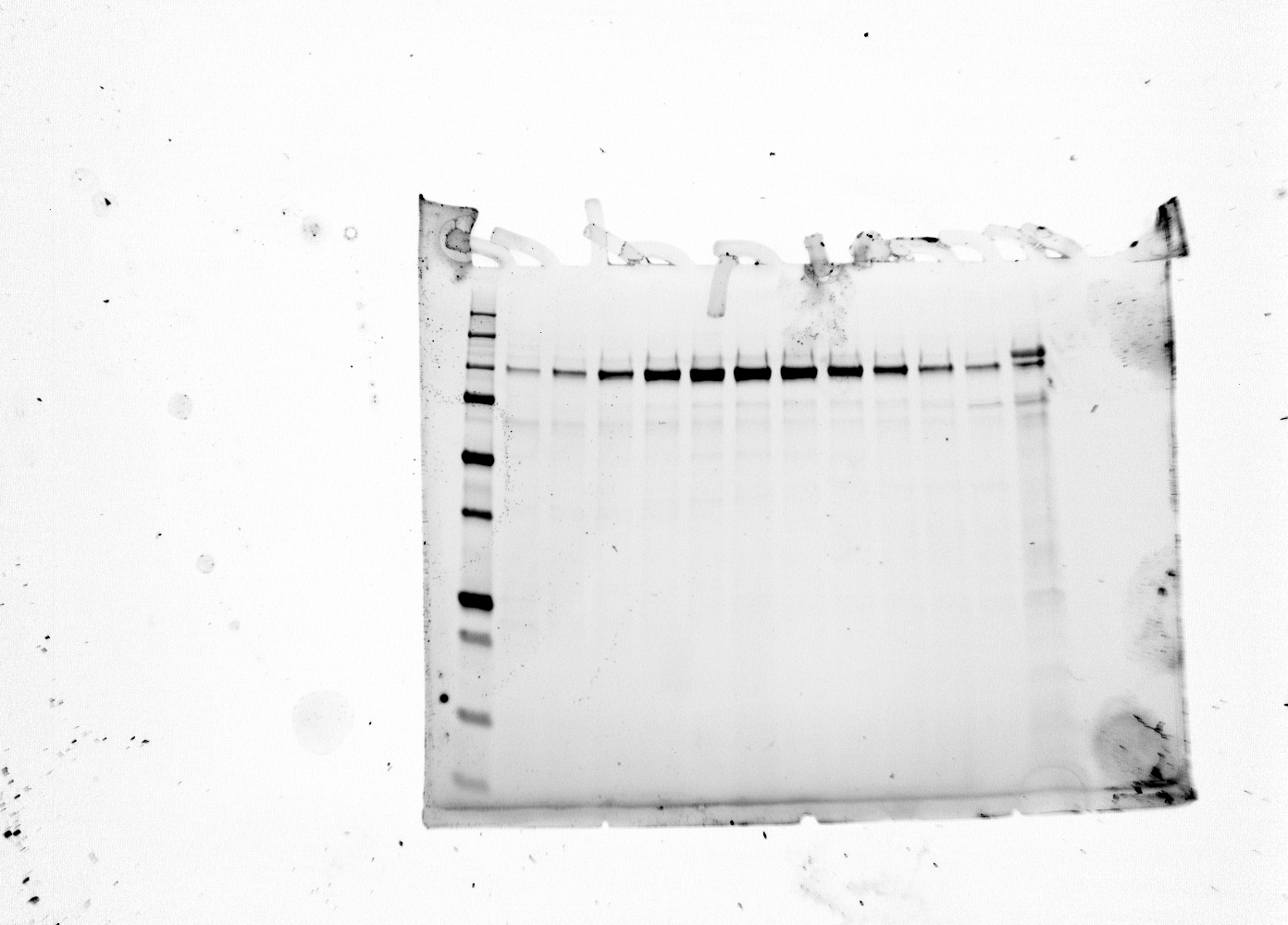

Supplement: Figure 1—figure supplement 1—source data 2. [file elife-60126-fig1-figsupp1-data2.zip › Figure 1-figure supplement 1-source data uncropped gels/Figure 1-figure supplement 1-source data 5.tif]

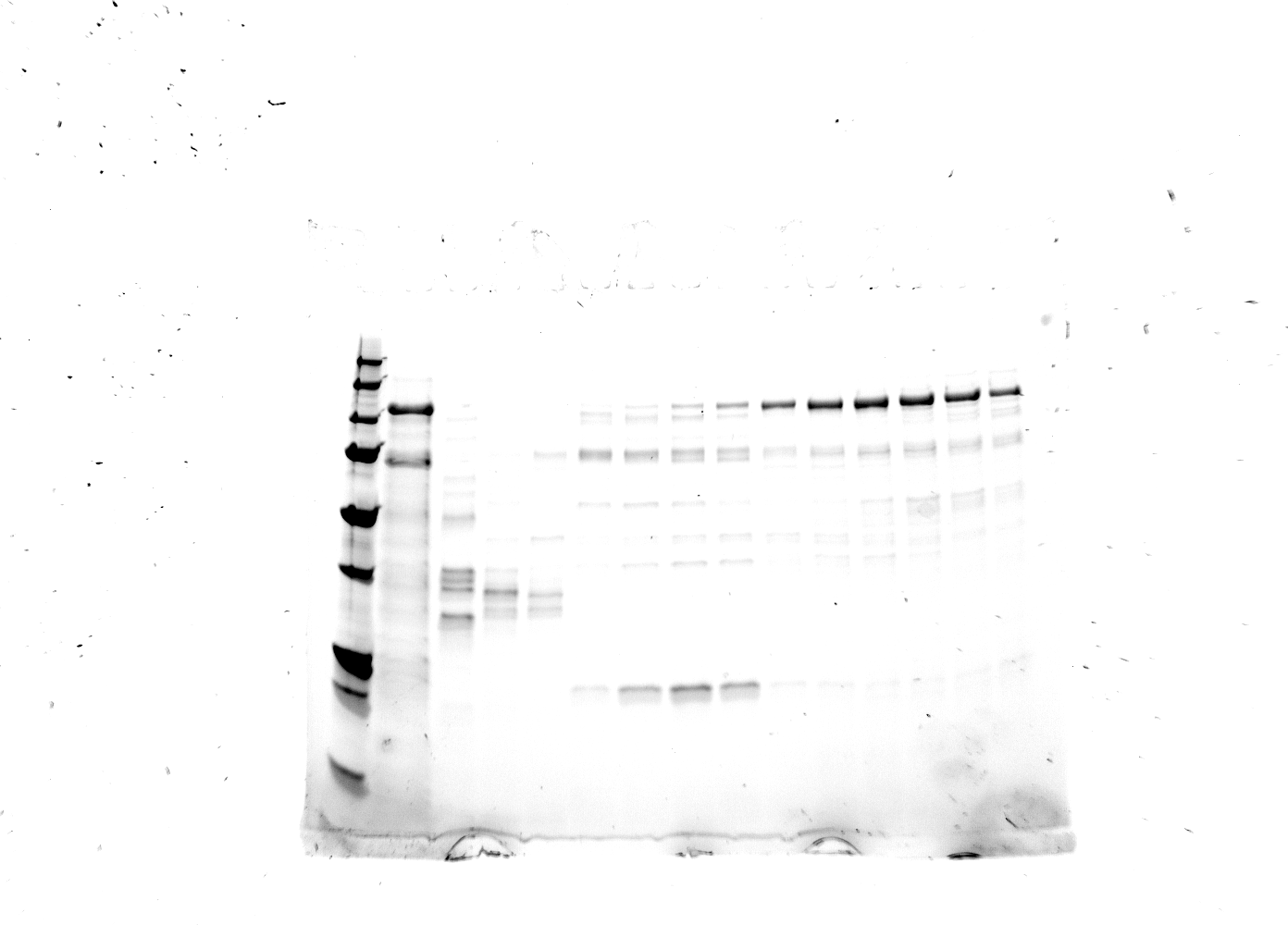

Supplement: Figure 1—figure supplement 1—source data 2. [file elife-60126-fig1-figsupp1-data2.zip › Figure 1-figure supplement 1-source data uncropped gels/Figure 1-figure supplement 1-source data 7.tif]

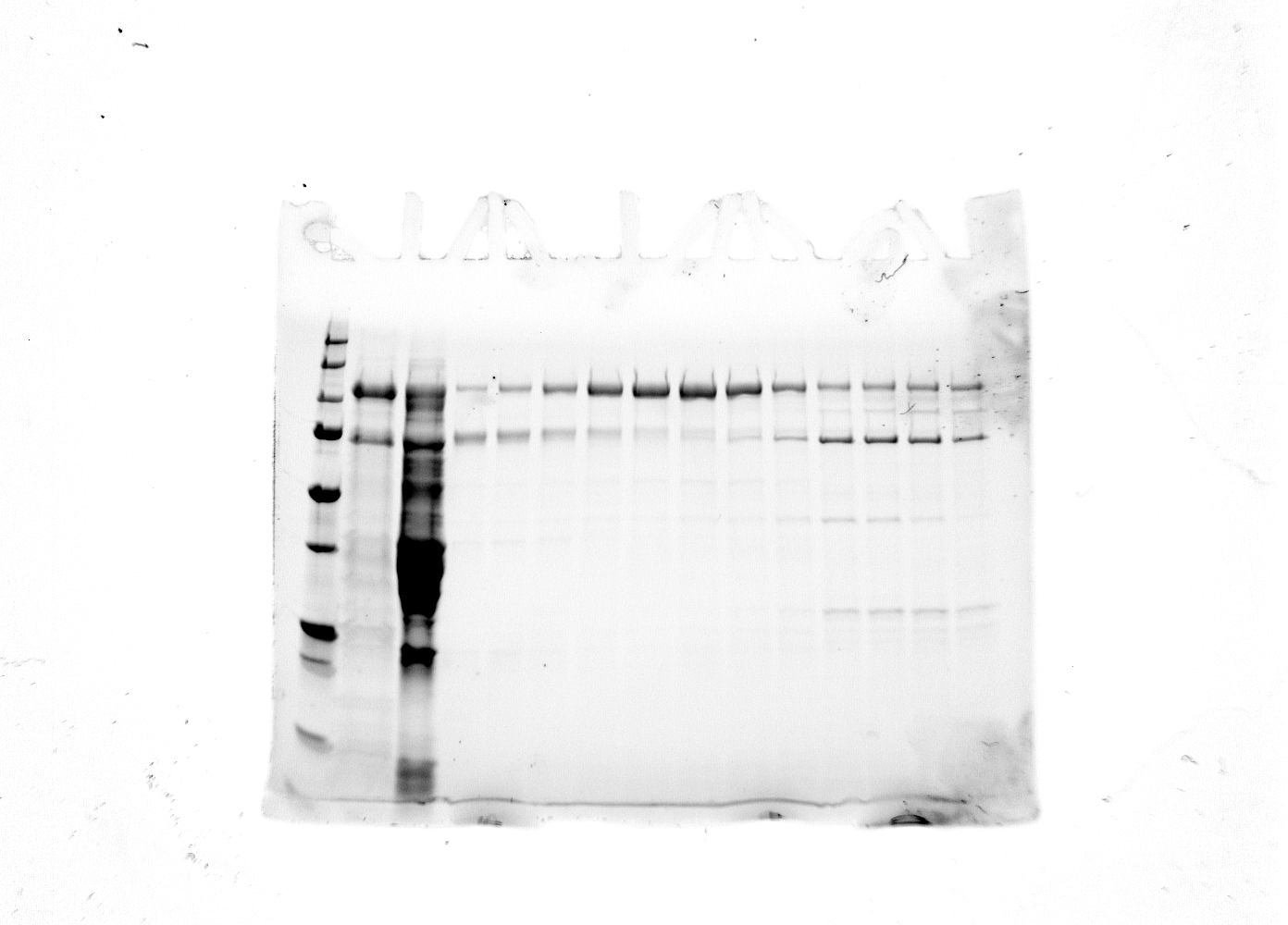

Supplement: Figure 1—figure supplement 1—source data 2. [file elife-60126-fig1-figsupp1-data2.zip › Figure 1-figure supplement 1-source data uncropped gels/Figure 1-figure supplement 1-source data 6.tif]

Figure 1-figure supplement 2-source data

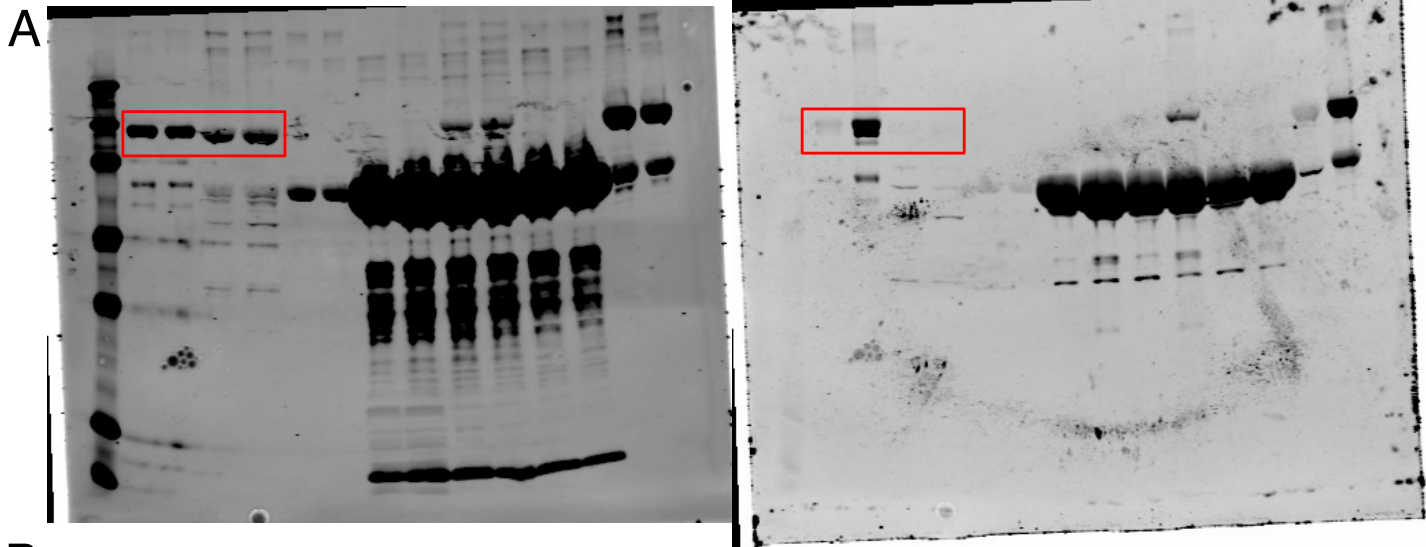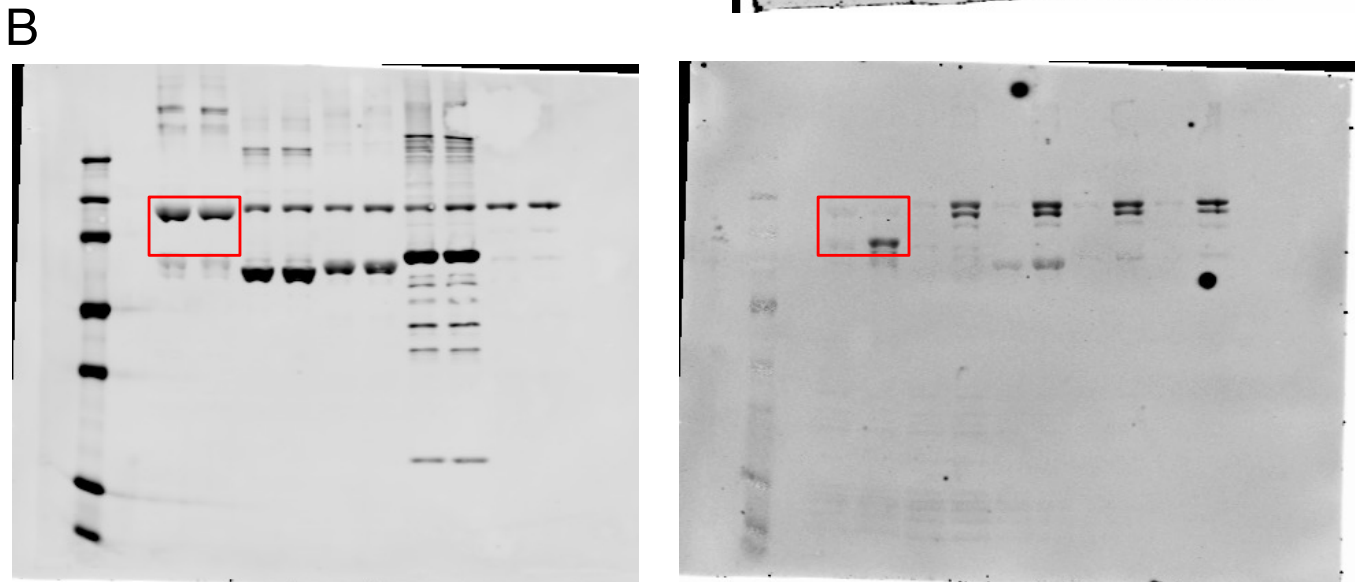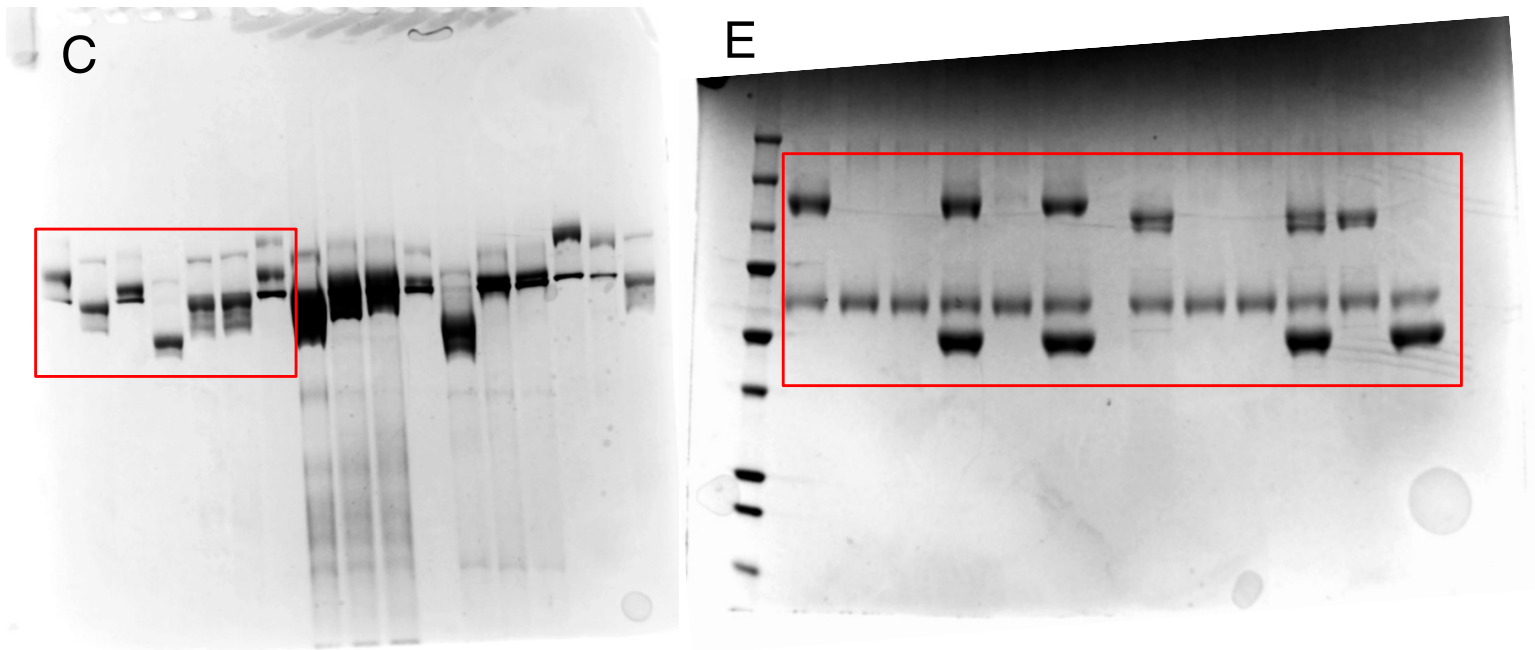

Supplement: Figure 1—figure supplement 2—source data 1. — The red box indicates how the blot of gel was cropped. [file elife-60126-fig1-figsupp2-data1.pdf]

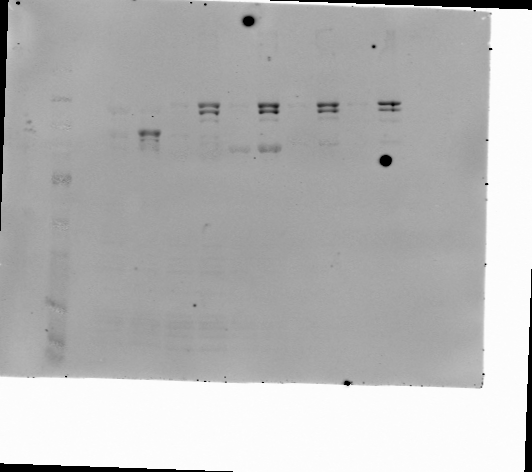

Supplement: Figure 1—figure supplement 2—source data 2. [file elife-60126-fig1-figsupp2-data2.zip › Figure 1-figure supplement 2-source data uncropped gels/Figure 1-figure supplement 2-source data 4.tif]

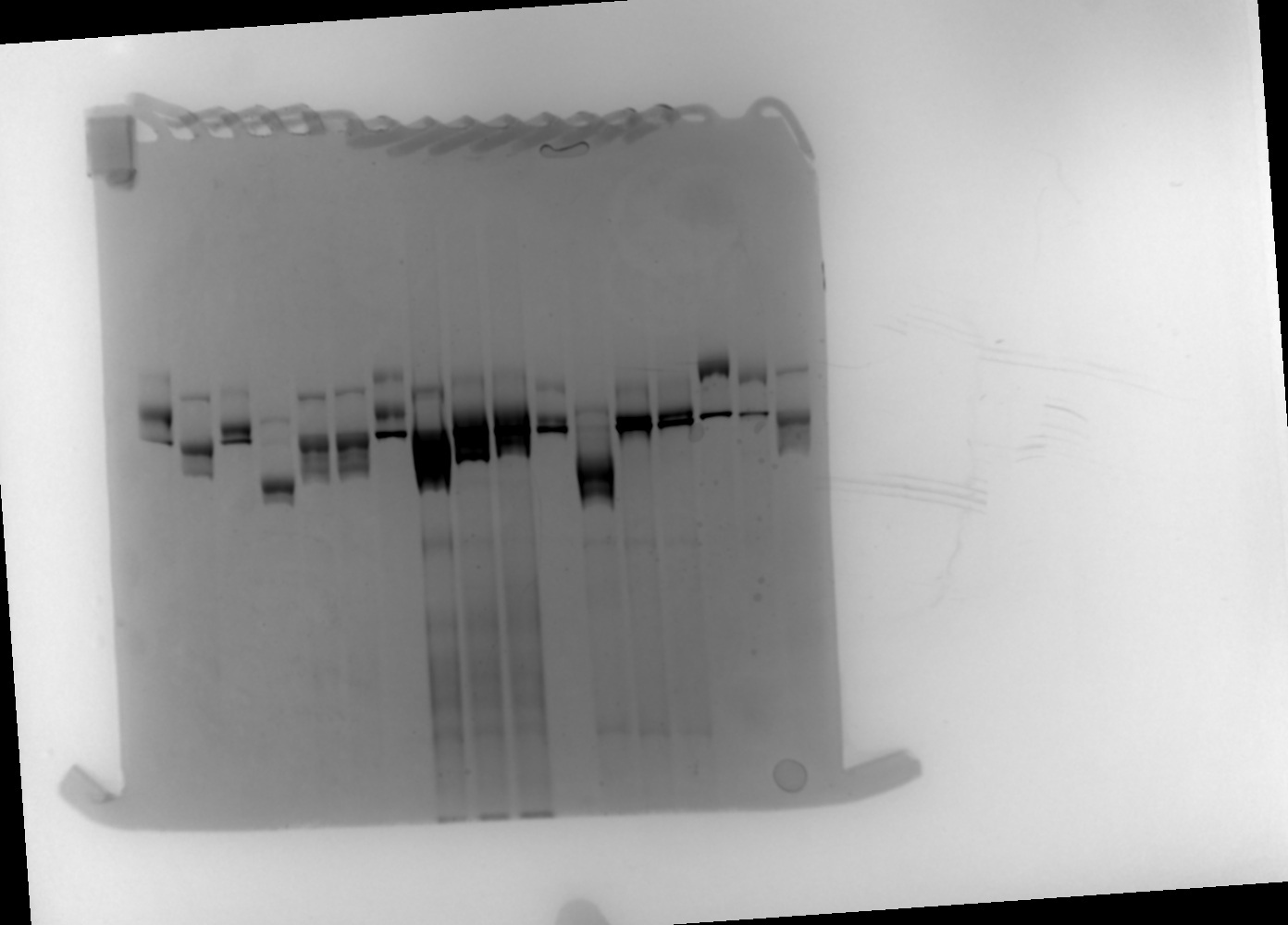

Supplement: Figure 1—figure supplement 2—source data 2. [file elife-60126-fig1-figsupp2-data2.zip › Figure 1-figure supplement 2-source data uncropped gels/Figure 1-figure supplement 2-source data 5.tif]

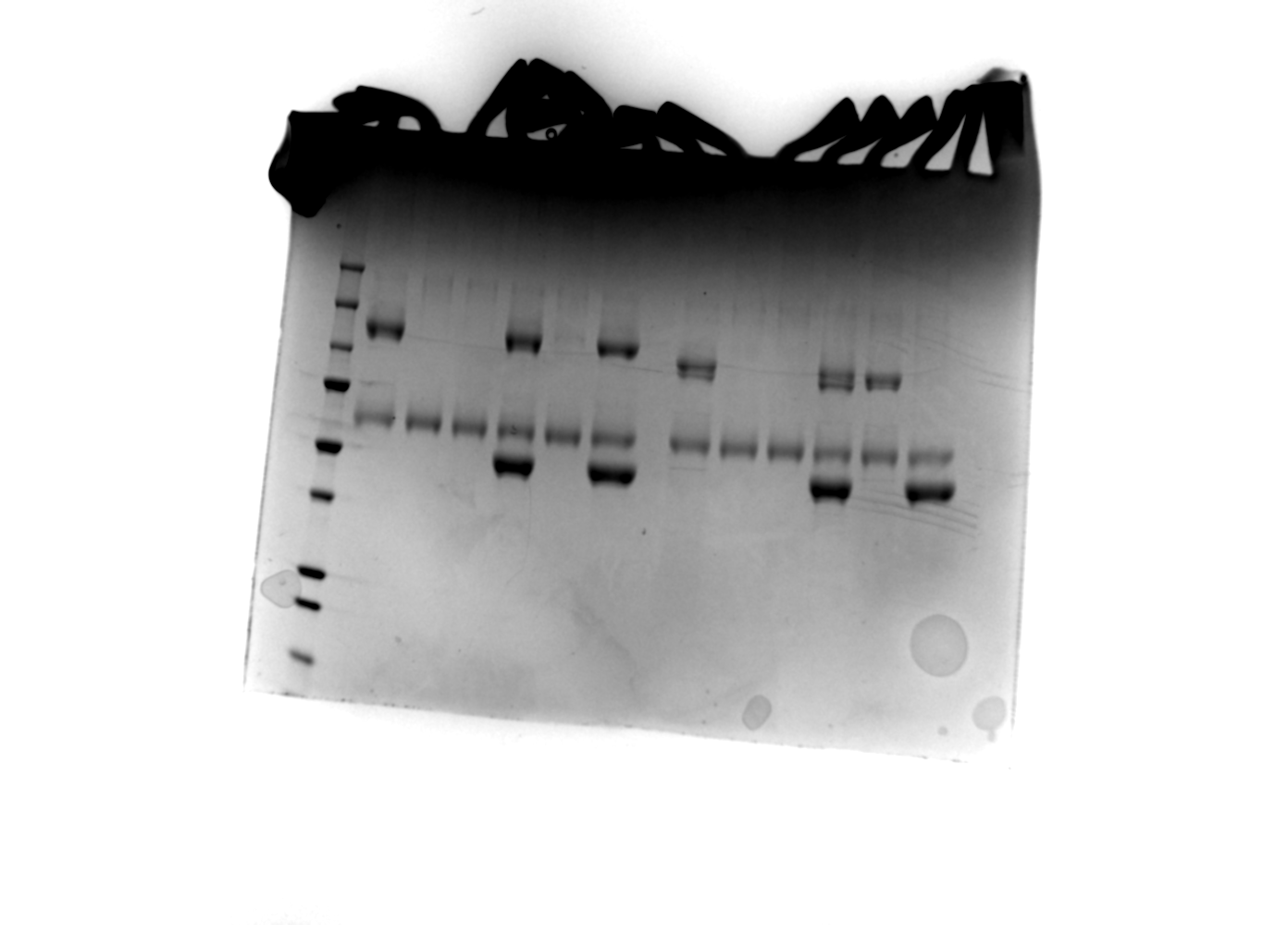

Supplement: Figure 1—figure supplement 2—source data 2. [file elife-60126-fig1-figsupp2-data2.zip › Figure 1-figure supplement 2-source data uncropped gels/Figure 1-figure supplement 2-source data 6.tif]

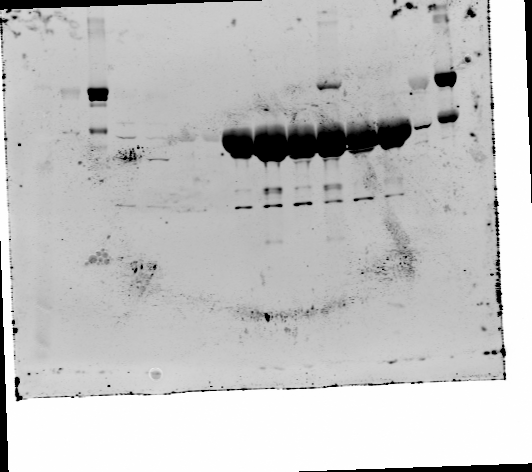

Supplement: Figure 1—figure supplement 2—source data 2. [file elife-60126-fig1-figsupp2-data2.zip › Figure 1-figure supplement 2-source data uncropped gels/Figure 1-figure supplement 2-source data 2.tif]

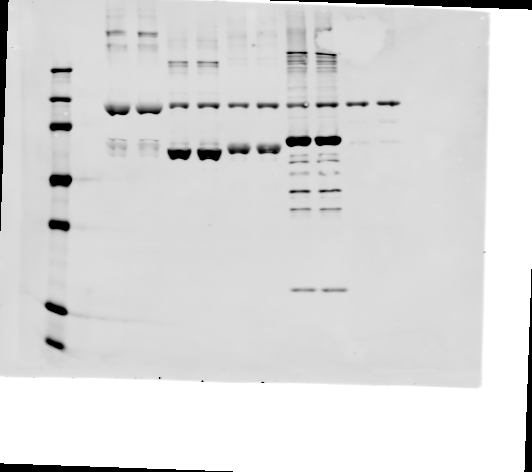

Supplement: Figure 1—figure supplement 2—source data 2. [file elife-60126-fig1-figsupp2-data2.zip › Figure 1-figure supplement 2-source data uncropped gels/Figure 1-figure supplement 2-source data 3.tif]

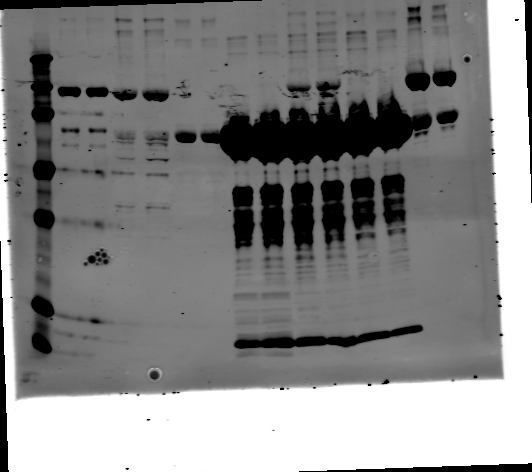

Supplement: Figure 1—figure supplement 2—source data 2. [file elife-60126-fig1-figsupp2-data2.zip › Figure 1-figure supplement 2-source data uncropped gels/Figure 1-figure supplement 2-source data 1.tif]

Figure 2-source data

A

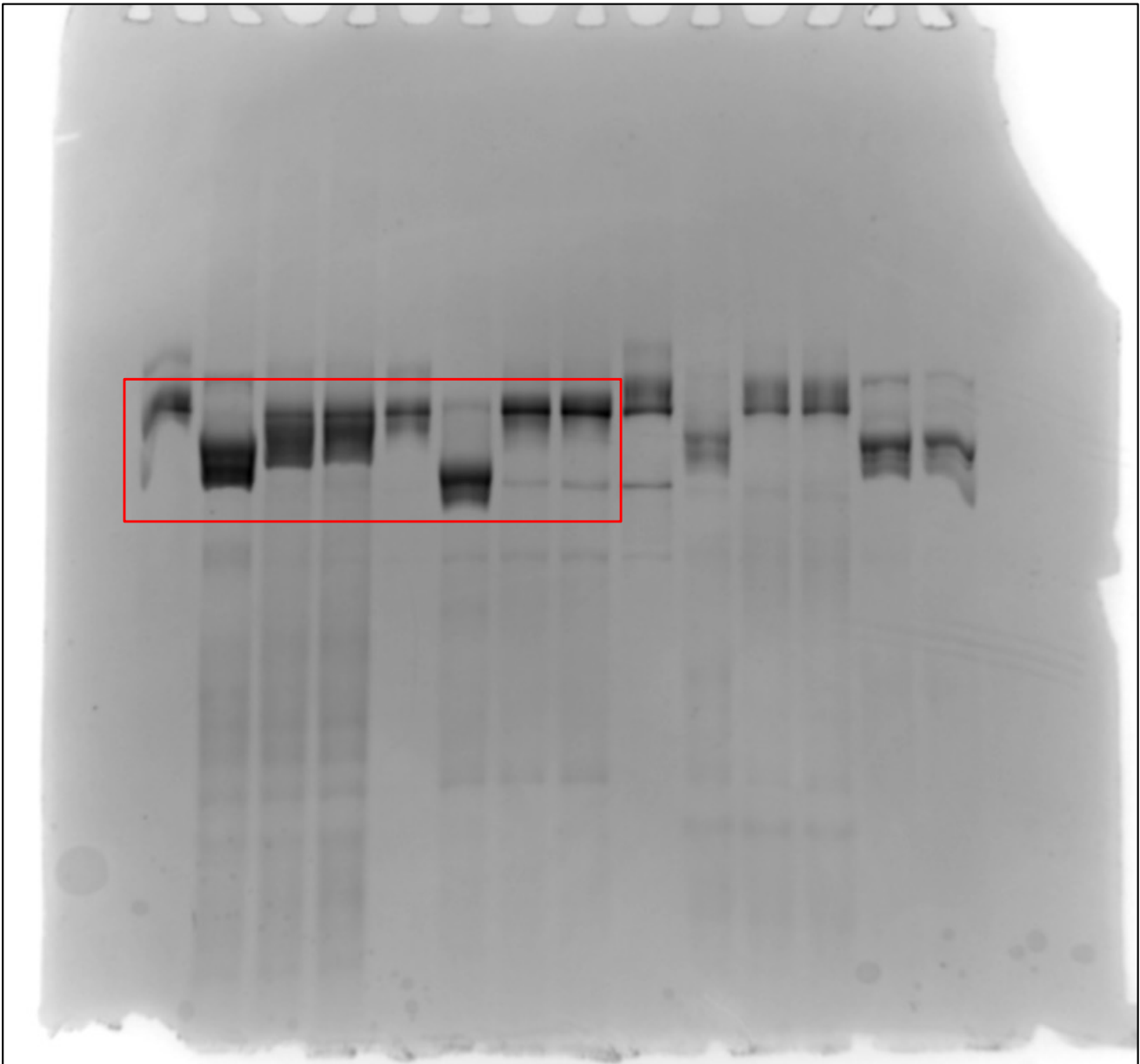

Supplement: Figure 2—source data 1. — The red box indicates how the gel was cropped. [file elife-60126-fig2-data1.pdf]

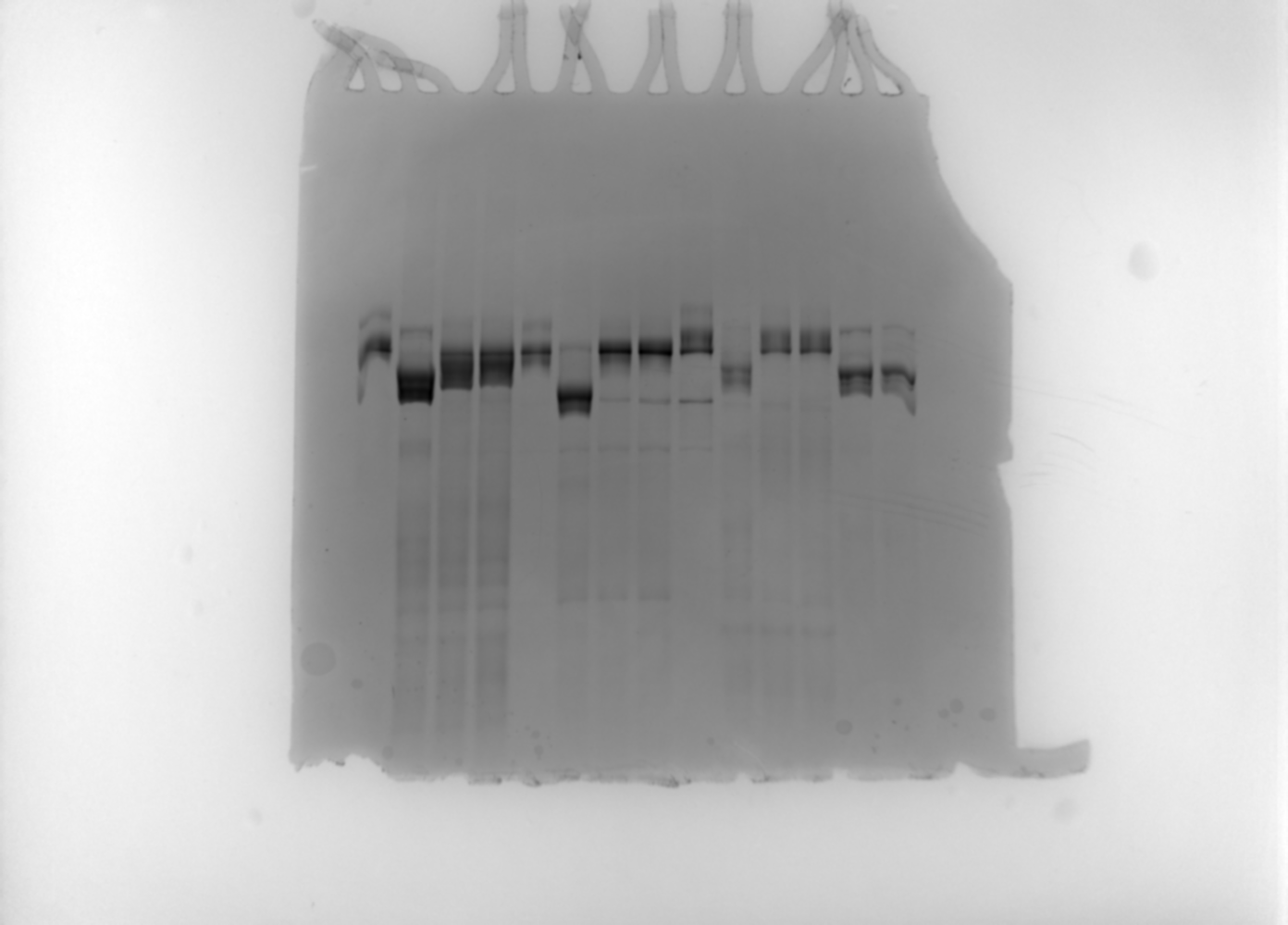

Supplement: Figure 2—source data 2. [file elife-60126-fig2-data2.zip › Figure 2-source data uncropped gel/Figure 2-source data uncropped gel.tif]

Figure 5-source data

B

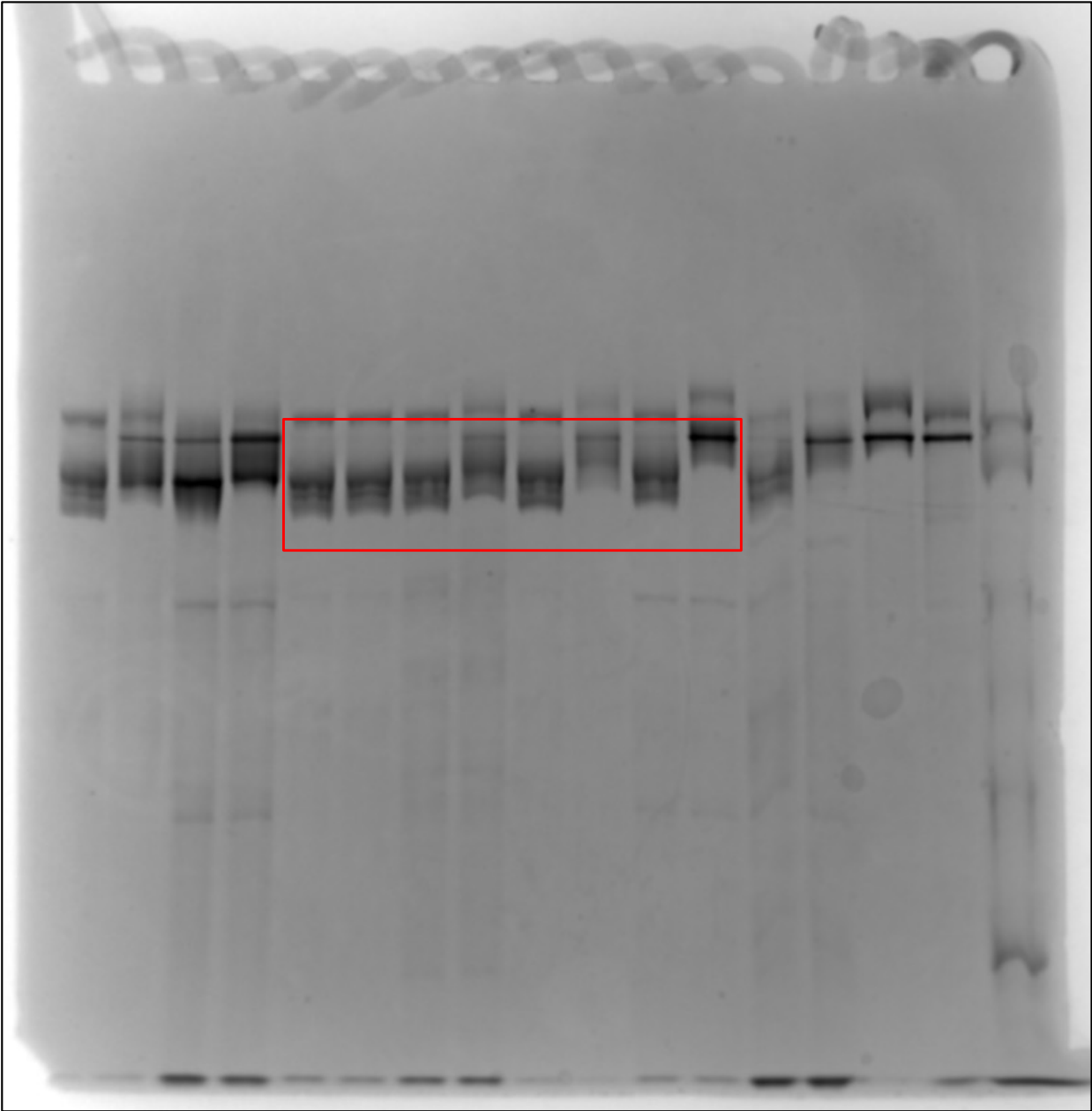

G

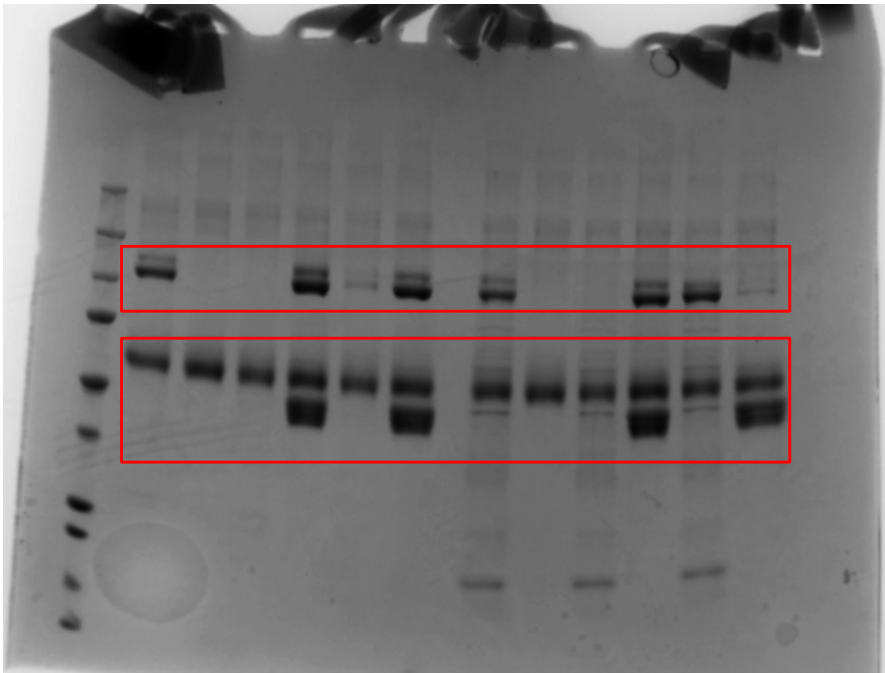

Supplement: Figure 5—source data 1. — The red box indicates how the gel was cropped. [file elife-60126-fig5-data1.pdf]

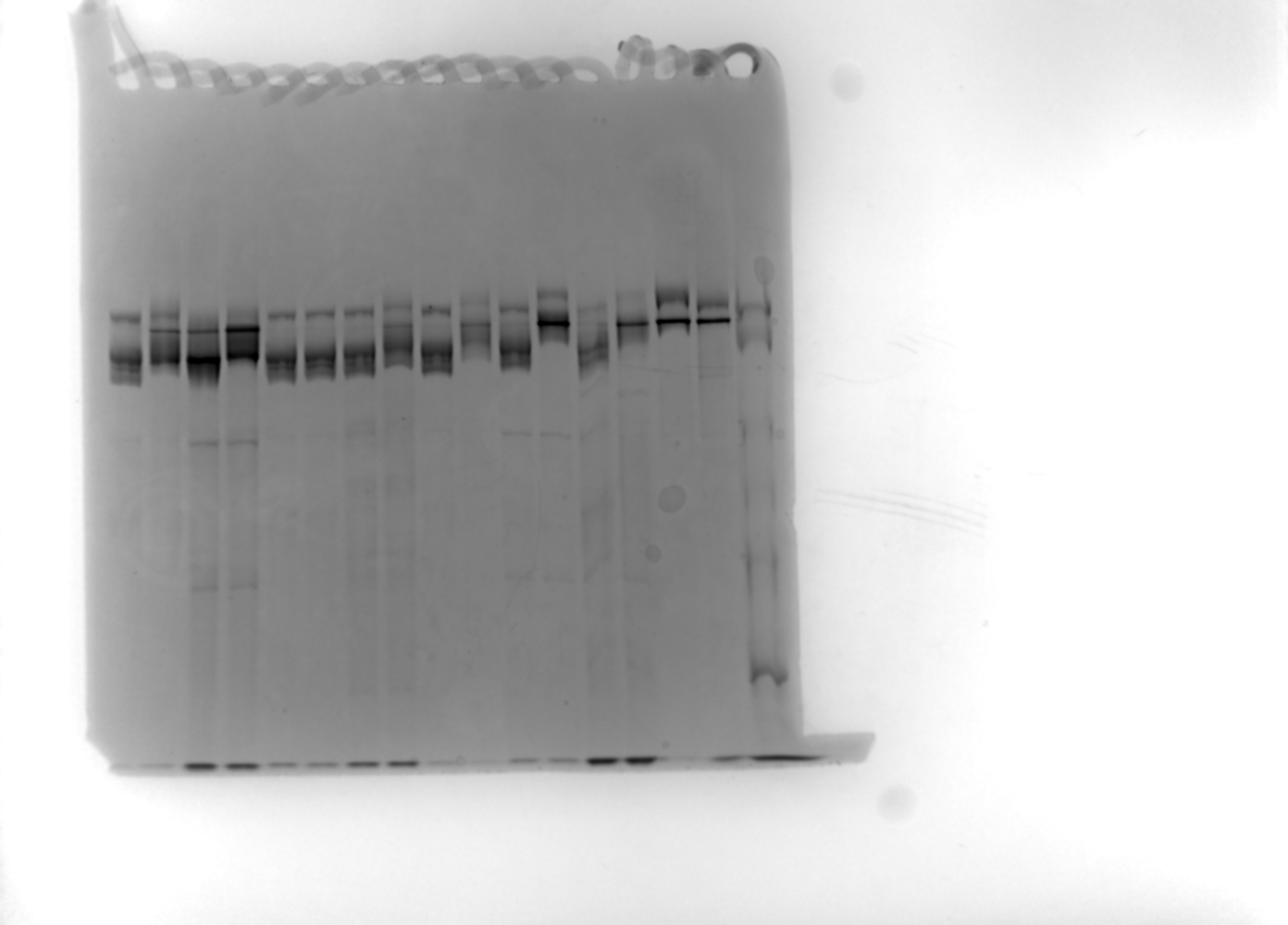

Supplement: Figure 5—source data 2. [file elife-60126-fig5-data2.zip › Figure 5-source data uncropped gels/Figure 5-source data 2.tif]

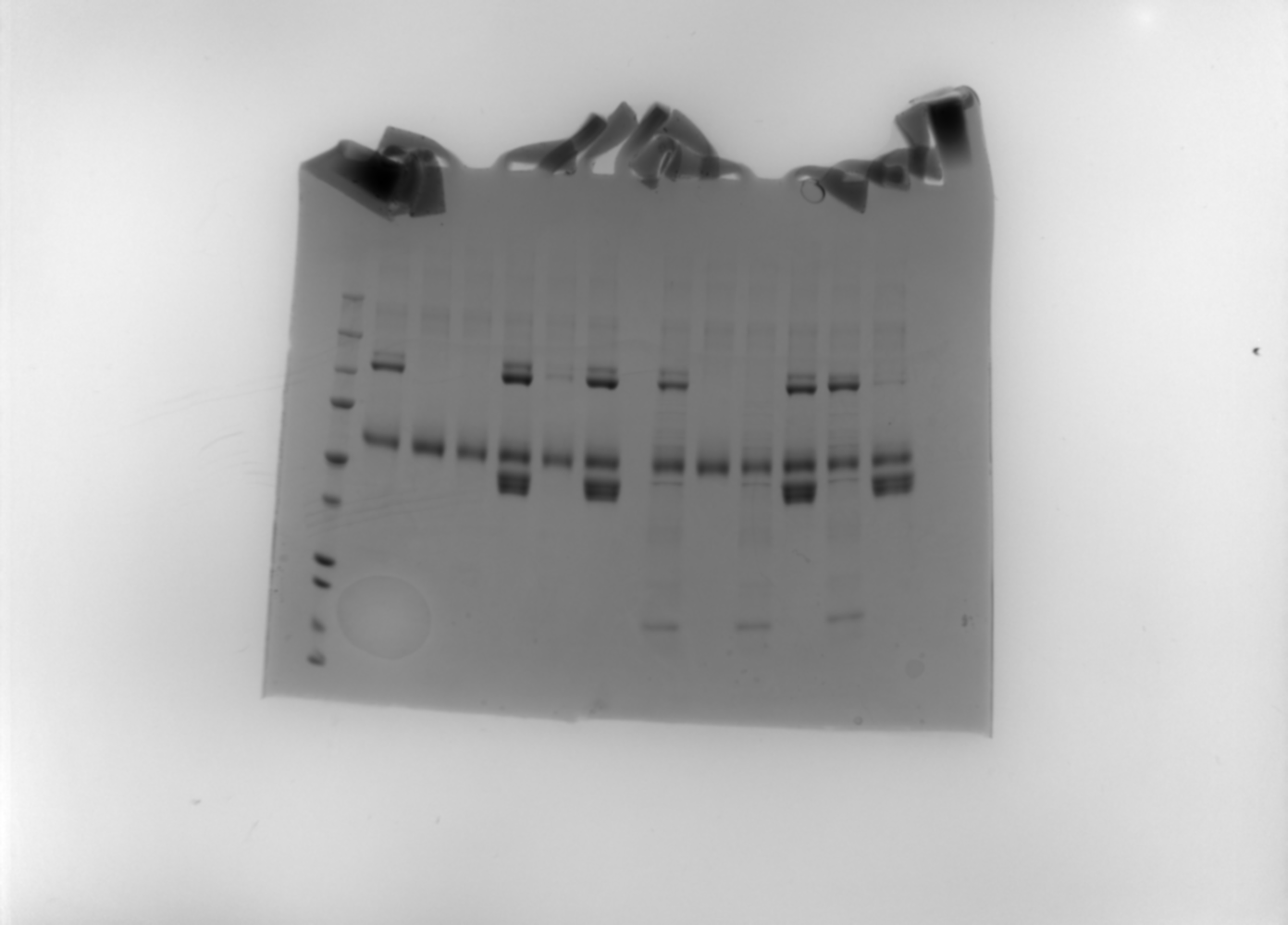

Supplement: Figure 5—source data 2. [file elife-60126-fig5-data2.zip › Figure 5-source data uncropped gels/Figure 5-source data 3.tif]

Figure 5-figure supplement 1-source data

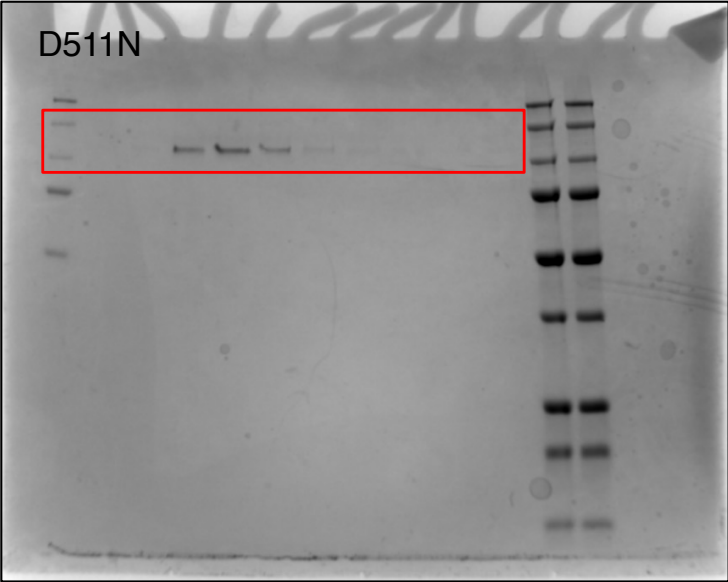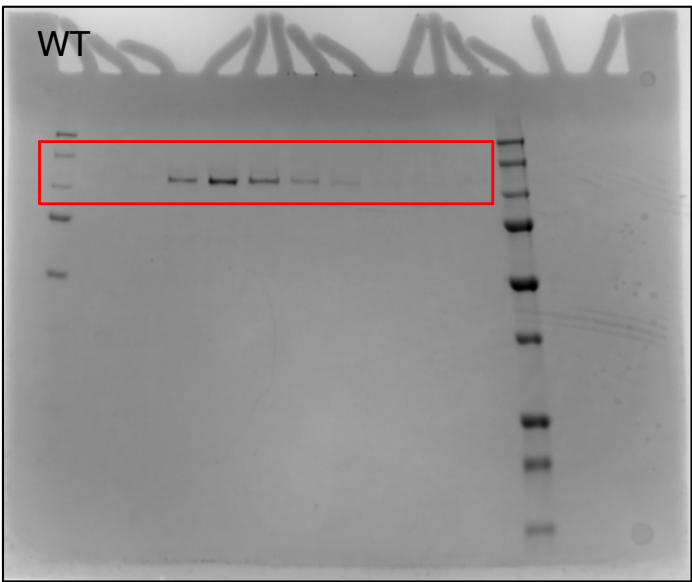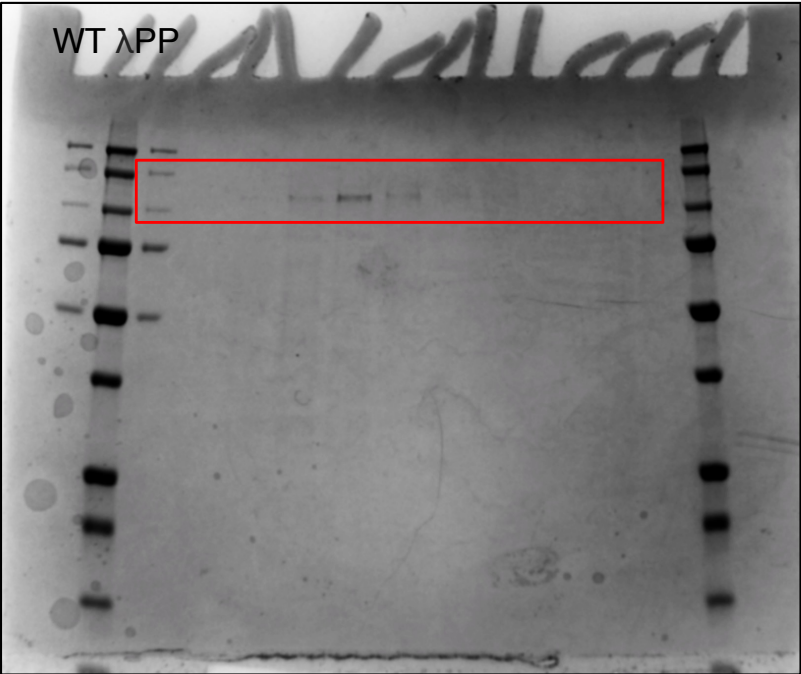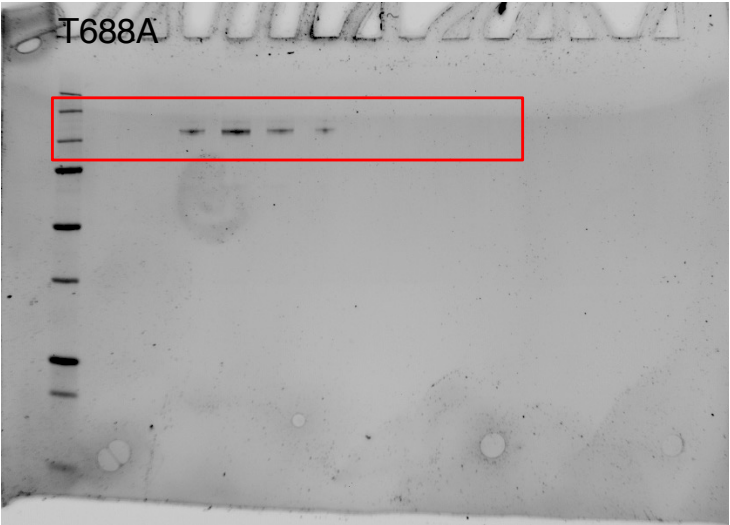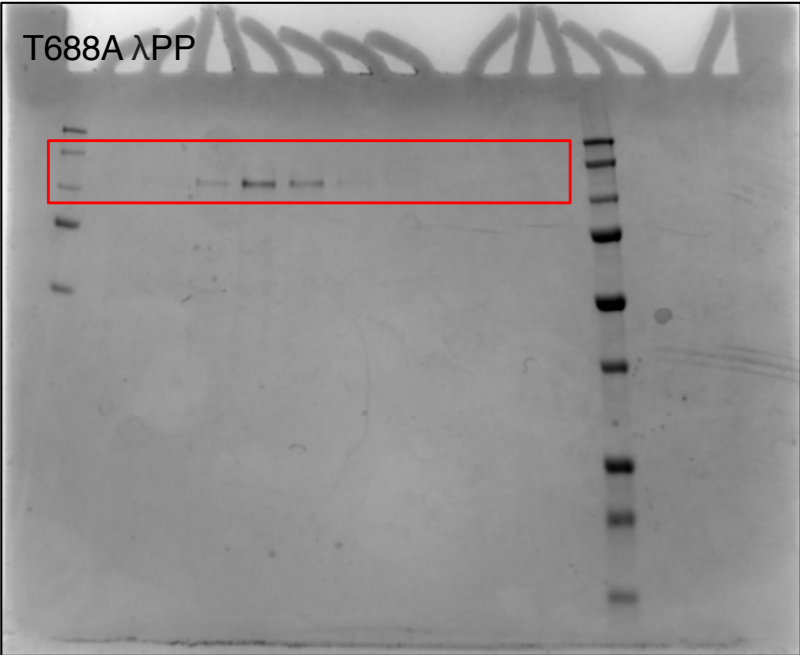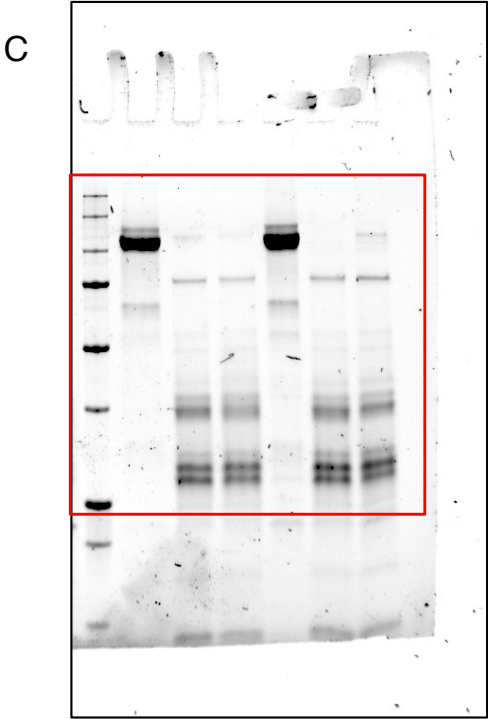

Supplement: Figure 5—figure supplement 1—source data 1. — The red box indicates how the gel was cropped. [file elife-60126-fig5-figsupp1-data1.pdf]

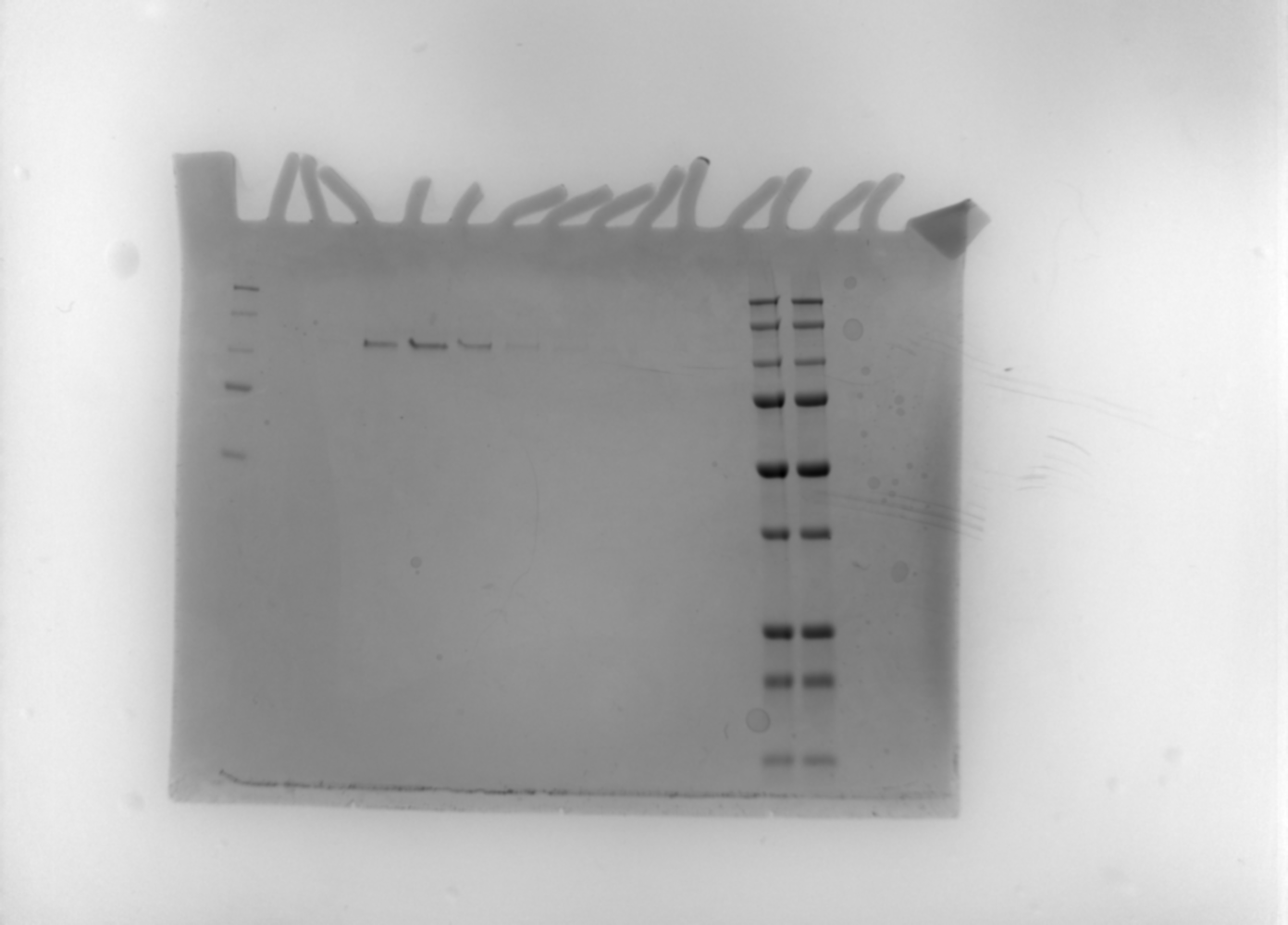

Supplement: Figure 5—figure supplement 1—source data 2. [file elife-60126-fig5-figsupp1-data2.zip › Figure 5-figure supplement 1-source data/Figure 5-figure supplement 1-source data 1.tif]

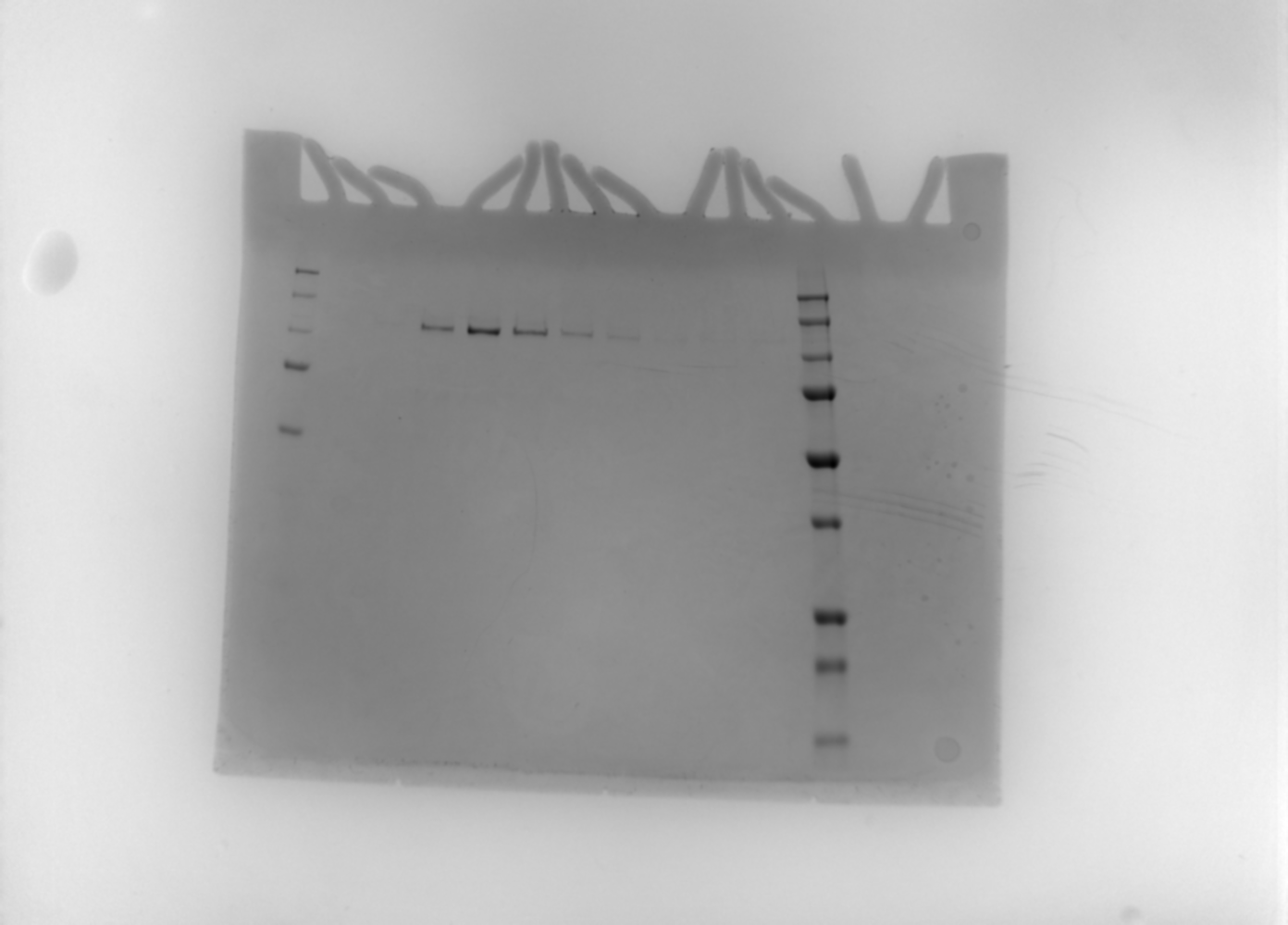

Supplement: Figure 5—figure supplement 1—source data 2. [file elife-60126-fig5-figsupp1-data2.zip › Figure 5-figure supplement 1-source data/Figure 5-figure supplement 1-source data 2.tif]

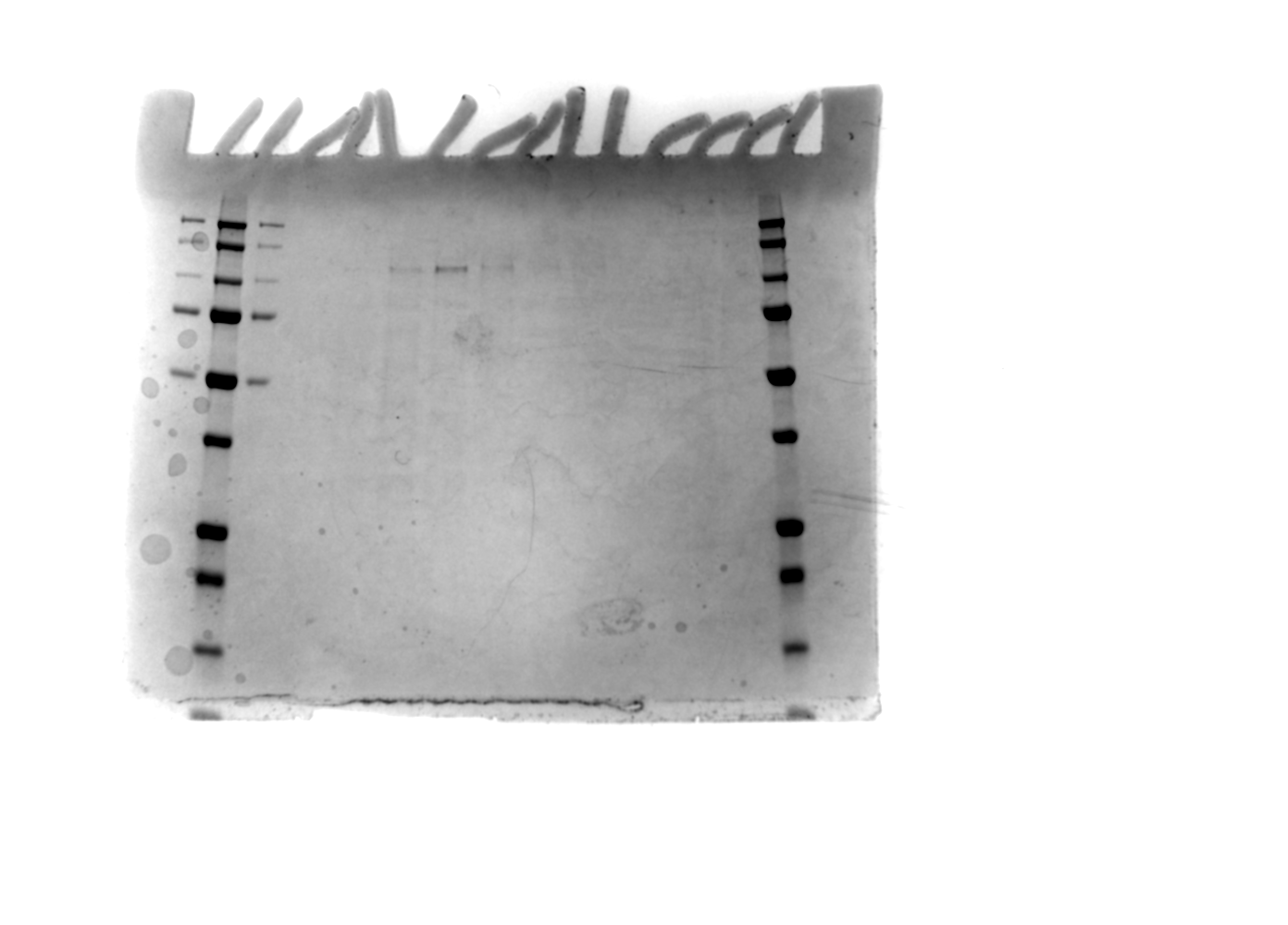

Supplement: Figure 5—figure supplement 1—source data 2. [file elife-60126-fig5-figsupp1-data2.zip › Figure 5-figure supplement 1-source data/Figure 5-figure supplement 1-source data 3.tif]

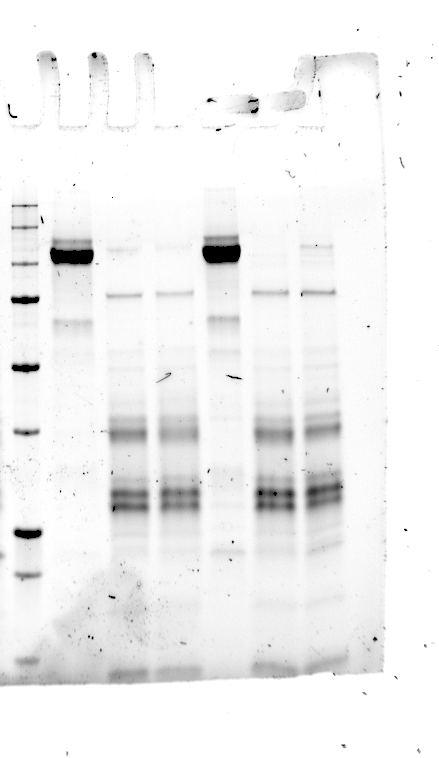

Supplement: Figure 5—figure supplement 1—source data 2. [file elife-60126-fig5-figsupp1-data2.zip › Figure 5-figure supplement 1-source data/Figure 5-figure supplement 1-source data 6.tif]

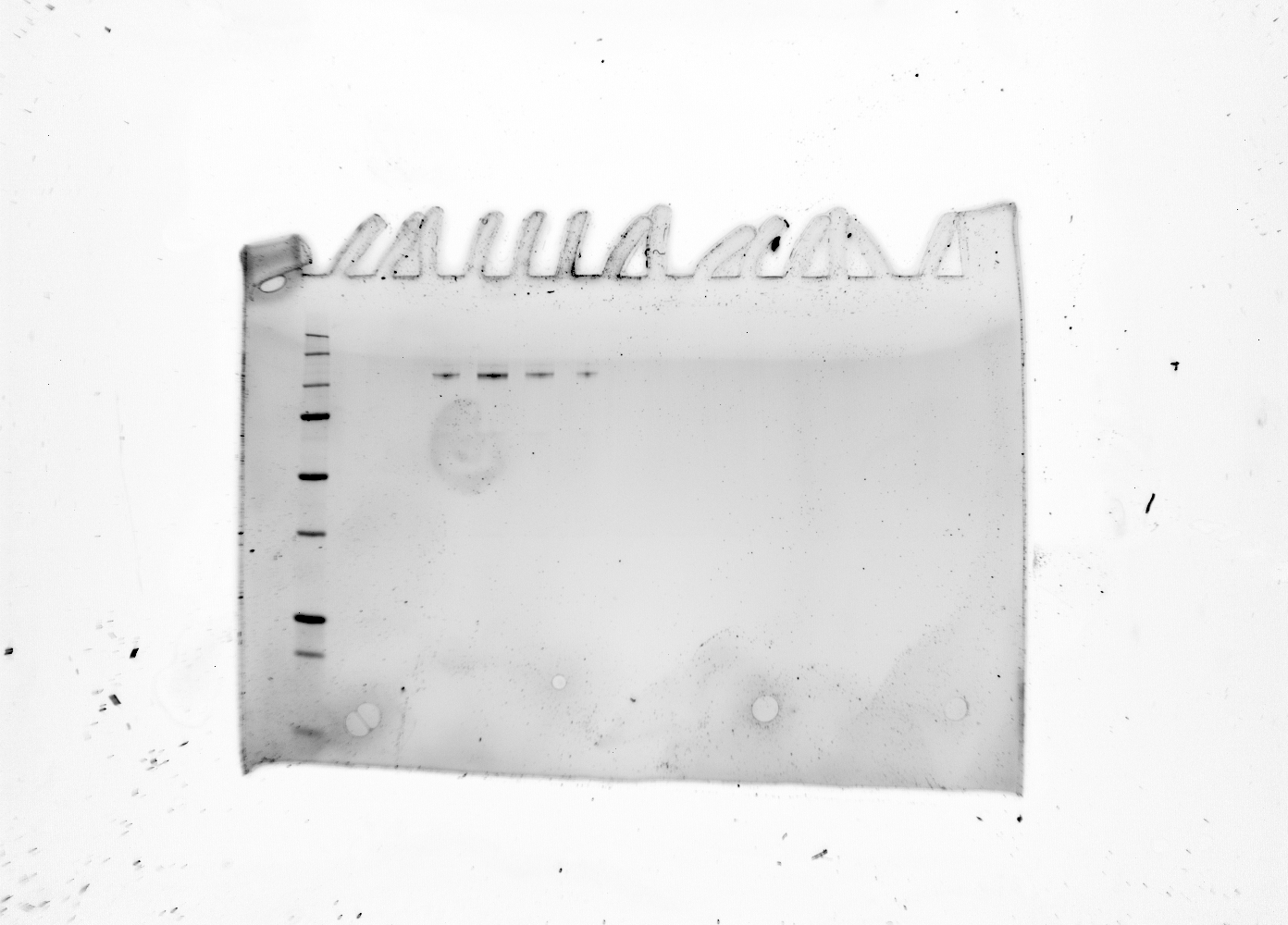

Supplement: Figure 5—figure supplement 1—source data 2. [file elife-60126-fig5-figsupp1-data2.zip › Figure 5-figure supplement 1-source data/Figure 5-figure supplement 1-source data 4.tif]

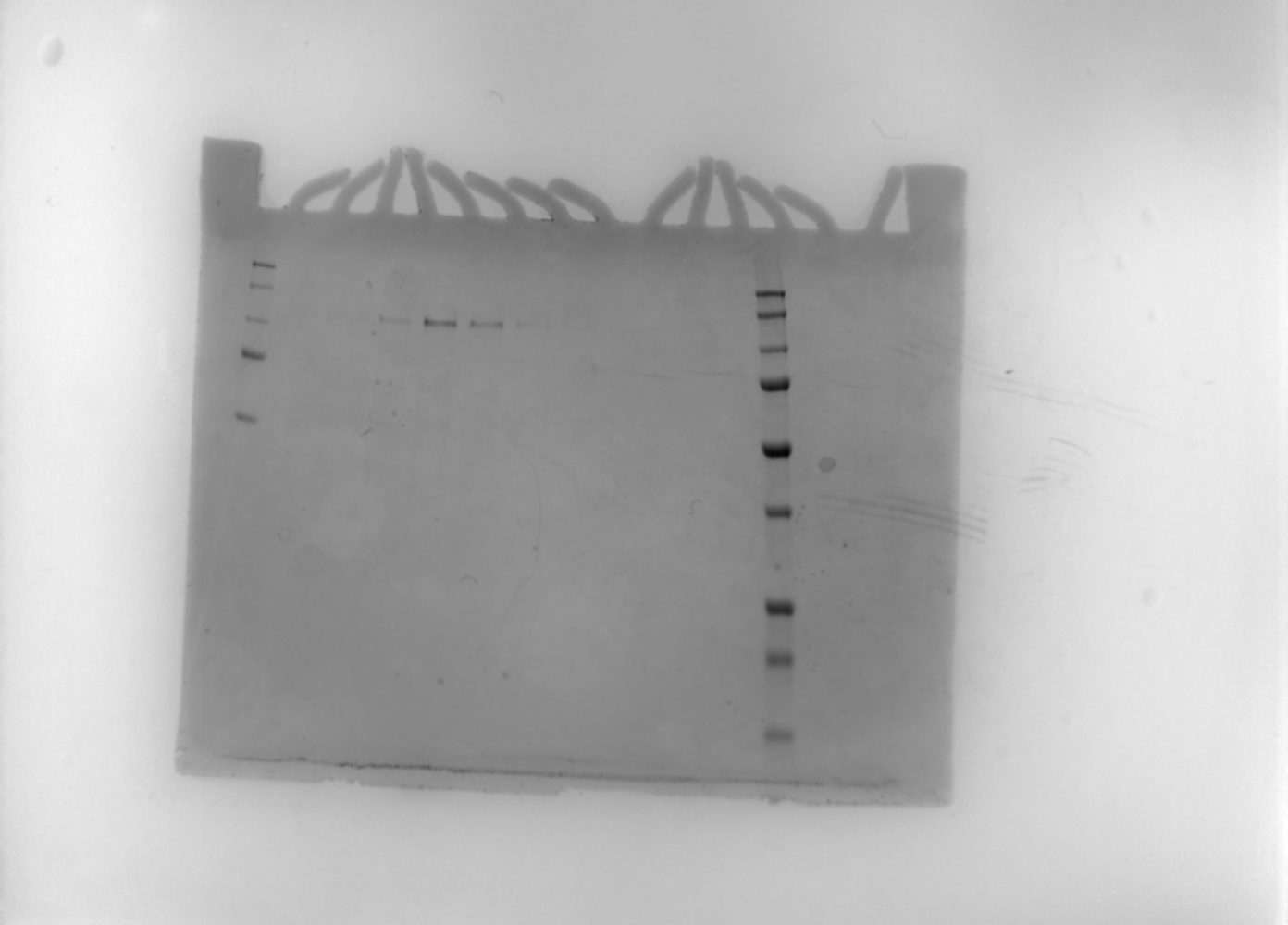

Supplement: Figure 5—figure supplement 1—source data 2. [file elife-60126-fig5-figsupp1-data2.zip › Figure 5-figure supplement 1-source data/Figure 5-figure supplement 1-source data 5.tif]
